# Serotonin neurons in the dorsal raphe nucleus encode reward signals

**DOI:** 10.1038/ncomms10503

**Published:** 2016-01-28

**Authors:** Yi Li, Weixin Zhong, Daqing Wang, Qiru Feng, Zhixiang Liu, Jingfeng Zhou, Chunying Jia, Fei Hu, Jiawei Zeng, Qingchun Guo, Ling Fu, Minmin Luo

**Affiliations:** 1 Graduate School of Peking Union Medical College, Chinese Academy of Medical Sciences, Beijing 100730, China; 2 National Institute of Biological Sciences, Zhongguancun Life Science Park 7 Science Park Road, Beijing 102206, China; 3 School of Life Sciences, Tsinghua University, Beijing 100084, China; 4 PTN Graduate Program, School of Life Sciences, Peking University, Beijing 100081, China; 5 Wuhan National Laboratory for Optoelectronics-Huazhong, Britton Chance Center for Biomedical Photonics, University of Science and Technology, Wuhan 430074, China

## Abstract

The dorsal raphe nucleus (DRN) is involved in organizing reward-related behaviours; however, it remains unclear how genetically defined neurons in the DRN of a freely behaving animal respond to various natural rewards. Here we addressed this question using fibre photometry and single-unit recording from serotonin (5-HT) neurons and GABA neurons in the DRN of behaving mice. Rewards including sucrose, food, sex and social interaction rapidly activate 5-HT neurons, but aversive stimuli including quinine and footshock do not. Both expected and unexpected rewards activate 5-HT neurons. After mice learn to wait for sucrose delivery, most 5-HT neurons fire tonically during waiting and then phasically on reward acquisition. Finally, GABA neurons are activated by aversive stimuli but inhibited when mice seek rewards. Thus, DRN 5-HT neurons positively encode a wide range of reward signals during anticipatory and consummatory phases of reward responses. Moreover, GABA neurons play a complementary role in reward processing.

## Abstract (web summary)

How neurons in the dorsal raphe nucleus (DRN) respond to reward related behaviours is not known. Here, Li and colleagues report that DRN serotoninergic neurons are phasically activated by rewards such as sex, food and sucrose, and tonically activated during reward anticipation, while GABAergic neurons respond to punishment.

## Graphical abstract

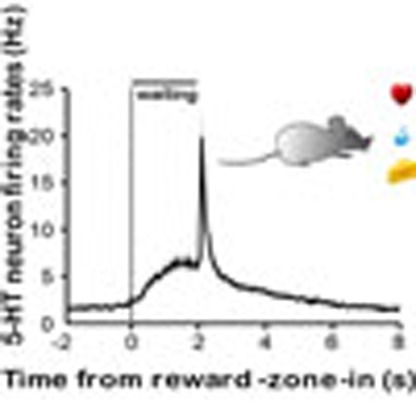

Various brain stations cooperate to organize reward-related behaviours. Best known is the midbrain ventral tegmental area, where dopamine neurons fire phasically to encode the discrepancy between the predicted and currently experienced reward^1^,^2^,^3^,^4^. More mysterious is the midbrain dorsal raphe nucleus (DRN). Its principle neurons project widely over the brain and strongly interconnect with several reward-related brain areas^5^,^6^,^7^,^8^. These neurons release the transmitter serotonin (5-hydroxytryptamine, 5-HT), which compels interest because 5-HT affects mood and drugs that increase 5-HT levels treat depression^9^,^10^.

Increasing evidences implicate that reward processing involves DRN neurons^11^. The 5-HT signalling system regulates feeding and social behaviours^12^,^13^,^14^. Slow and diffusive 5-HT signals may determine global reward states to modulate mood^15^. The DRN represents one of the most effective brain sites that drive electrical and optogenetic self-stimulation^16^,^17^,^18^,^19^. Surprisingly, the stimulation-evoked reward signal is mainly mediated by glutamate, although a majority of 5-HT neurons are glutamatergic and 5-HT also plays a role^17^. Optogenetic stimulation of 5-HT neurons also promotes waiting for expected reward, suggesting their role in the reward anticipatory behaviour^20^,^21^,^22^,^23^.

Knowing neuronal activity patterns can help clarify how the DRN contributes to reward processing. In primates and rodents, the activity of DRN neurons is correlated with arousal^24^, sensory cues^25^,^26^, motor activity^25^,^27^,^28^, reward values^29^,^30^,^31^, delay^20^, task progress^32^, aversive stimuli^31^,^33^ and the absence of reward^26^. The response diversity may reflect the heterogeneity of DRN neurons in morphology, location and neurotransmitter phenotypes^34^,^35^,^36^. It is challenging to precisely identify neuron types using electrophysiological criteria in extracellular recordings^37^,^38^. Using optogenetic tagging, two recent recordings revealed that reward-predicting cues activate approximately half of 5-HT neurons^17^,^39^. These two recordings, however, were performed from head-fixed mice that chronically experienced restraint stress. Moreover, the behavioural tasks was limited to classic conditioning, which trained mice to link an olfactory cue with the expected delivery of liquid reward^17^,^39^.

Many key questions remain. As food and sex are fundamental to animal survival and reproduction, how do DRN 5-HT neurons respond to these important natural rewards? Moreover, are these cells activated only when a well-trained animal is waiting for expected rewards? Alternatively, can they be also activated after an animal receives the reward, either expected or unexpected? As GABA neurons comprise a major neuron population in the DRN^6^,^40^,^41^, do they behave differently from 5-HT neurons? In the present study, we tackled these questions using fibre photometry of Ca^2+^ signals and electrophysiological recordings of action potential firing from genetically identified 5-HT and GABA neurons in freely behaving mice. Recording from freely behaving animals is essential for studying the responses to food intake and social interactions. It also avoids restraint-associated inescapable stress that may change the activity of DRN neurons and 5-HT signalling^42^,^43^.

Our recordings reveal that DRN 5-HT neurons are rapidly activated when a mouse voluntarily seeks and acquires sucrose, food, sex and social interaction. Moreover, 5-HT neurons are activated by surprising delivery of appetitive but not aversive stimuli. For a mouse that has been trained to wait for sucrose reward, 5-HT neurons encode reward waiting and acquisition through a tonic-then-phasic activation mode. Finally, GABA neurons in the DRN are suppressed during reward seeking but activated by footshock. These results indicate that DRN 5-HT neurons and GABA neurons respond complementarily when an animal expects and receives various rewards, suggesting that the dorsal raphe serves as an important reward-processing station in parallel to the midbrain dopamine centre.

## Results

### Food and social rewards activate DRN 5-HT neurons

**Figure 1:**
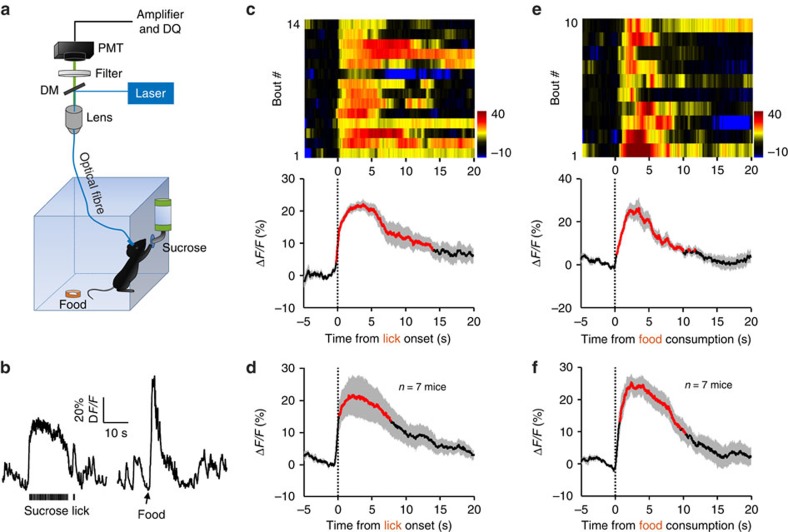
Sucrose lick and food intake increase Ca^2+^ signals of DRN 5-HT neurons. (**a**) Schematic of the fibre photometry setup. Ca^2+^ transients were recorded from GCaMP6-expressing DRN 5-HT neurons of *SERT-Cre* mice that had free access to sucrose solution and food pellets in a test chamber. DM, dichroic mirror; PMT, photomultiplier tube. (**b**) Raw traces of GCaMP6 fluorescence changes that were associated with sucrose lick and food intake. Δ*F/F* represents change in fluorescence from the mean level before the task. (**c**) Ca^2+^ signals associated with bouts of sucrose lick in a behavioural session. Upper panel, the heatmap illustration of Ca^2+^ signals aligned to the initiation of sucrose licking bouts. Each row plots one bout and a total of 14 bouts are illustrated. Colour scale at the right indicates Δ*F/F*. Lower panel, the peri-event plot of the average Ca^2+^ transients and lick frequency. Thick lines indicate mean and shaded areas indicate s.e.m. Red segments indicate statistically significant increase from the baseline (*P*<0.05; permutation test). (**d**) Mean Ca^2+^ transients associated with sucrose lick for the entire test group (*n*=7 mice). (**e**,**f**) Ca^2+^ transients elicited by food intake (*n*=7 mice). Data from ten bouts are plotted in **e**. Same conventions as in **c** and **d**.

We used fibre photometry to record Ca^2+^ signals from 5-HT neurons of behaving mice (Fig. 1a). Similar setups accomplished stable recordings from specific neuron populations in the striatum, cortex and ventral tegmental area^44^,^45^,^46^. Following stereotaxic infusion of the Cre-dependent adeno-associated virus AAV-DIO-GCaMP6m into the DRN of *SERT-Cre* transgenic mice (henceforth referred to as *SERT*-DRN^GCaMP6^ mice)^47^,^48^, the calcium indicator GCaMP6 was expressed with >98.7% accuracy and efficiency in neurons expressing tryptophan hydroxylase 2 (Supplementary Fig. 1a), the rate-limiting enzyme for central 5-HT synthesis^49^. We implanted a small optical fibre (230 μm diameter) with its tip into the DRN for chronic recordings of GCaMP fluorescence changes (Supplementary Fig. 1b).

We first examined GCaMP signals when *SERT*-DRN^GCaMP6^ mice sought and acquired sucrose solution. A water-deprived mouse received sucrose by licking a nozzle that was linked to a contact lickometer (Fig. 1a). The mouse completed 14 licking bouts within a 20-min behaviour session. Sucrose lick reliably increased GCaMP fluorescence across the behavioural bouts for this mouse (Fig. 1b,c). Multivariate permutation tests revealed statistically significant GCaMP signals in six out of seven test mice (*P*<0.05; Fig. 1d and Supplementary Fig. 1c). Post-mortem examination revealed that the six responsive recording sites were located in the centre DRN, whereas the non-responsive site was located in the edge of the DRN where GCaMP6 expression was weak (Supplementary Fig. 1b). For the six recordings in the centre DRN, the average signal peak (Δ*F/F*) was 23.6±4.4% (mean±s.e.m., *n*=6 mice). The increase of fluorescence signals was tightly coupled to the lick onset and lasted for approximately the same duration of licking (12.8±1.1 s for GCaMP signals versus 14.7±2.1 s for lick, mean±s.e.m., *n*=6 mice).

Using fibre photometry, we next measured GCaMP responses to food intake. A food-deprived mouse retrieved a small food pellet that was manually delivered to a tray (Fig. 1a). The events of food intake were identified by offline video scoring and time-locked GCaMP signals were segmented and aligned (Fig. 1b,e). Similar to sucrose lick, the events of food intake were reliably associated with significant increase of GCaMP signals (Δ*F/F*=20.8±1.4%; duration of significant signals=12.1±2.7 s; mean±s.e.m., *P*<0.05, permutation test, *n*=7 mice; Fig. 1e,f and Supplementary Fig. 1d). Mice that expressed enhanced membrane green fluorescent protein (EmGFP) did not show any clear fluorescence change following sucrose lick or food intakes (*n*=6 *SERT*-DRN^EmGFP^ mice; Supplementary Fig. 1e,f), indicating that the signals in *SERT*-DRN^GCaMP6^ mice were Ca^2+^ in nature and not motion artefacts.

**Figure 2:**
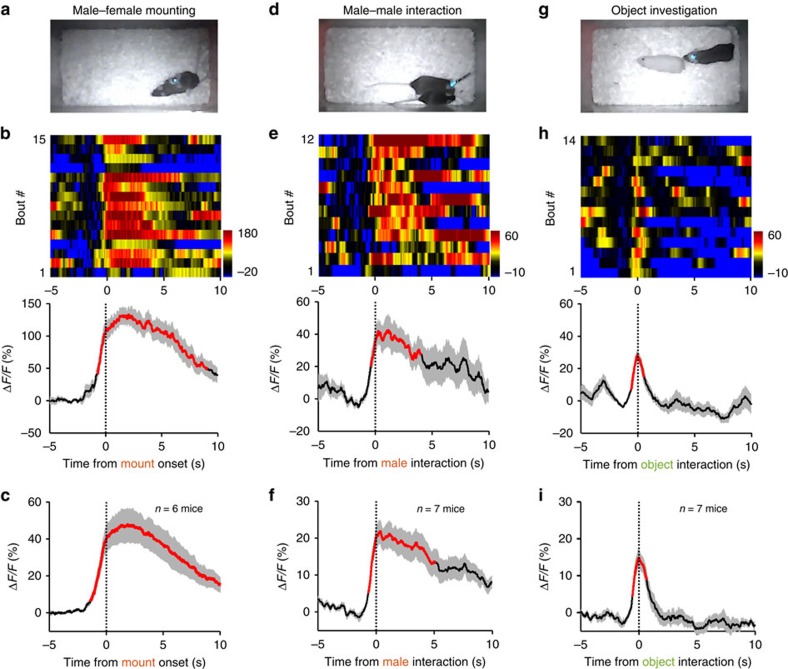
Social rewards activate DRN 5-HT neurons. (**a**–**c**) Ca^2+^ signals associated with mating behaviour of male mice. A SERT-DRN^GCaMP6^ male mouse mounted a female following the introduction of the receptive female into the home cage of the male (**a**). Heatmap of Ca^2+^ transients and average plots show that mounting a female resulted in significant increase of Ca^2+^ across multiple behavioural bouts of a male mouse (**b**) and for the entire test group (**c**, *n*=6 mice). Thick lines indicate mean and shaded areas indicate s.e.m. Red lines indicate significant increase (*P*<0.05; permutation test). (**d**–**f**) Ca^2+^ signals associated with male–male interaction. GCaMP signals were recorded when a test male performed chemoinvestigation of an intruder male. Same conventions as in **a**–**c**. *n*=7 SERT-DRN^GCaMP6^ male mice in **f**. (**g**–**i**) Ca^2+^ signals associated with investigation of a neutral object. An inanimate dummy mouse was introduced into the home cage of a test male (**g**). *n*=7 SERT-DRN^GCaMP6^ male mice in **i**.

We then investigated how DRN 5-HT neurons respond to social rewards. We applied fibre photometry to record from the DRN of a male mouse in its home cage and simultaneously videotaped its interaction with another mouse. The test male often initiated mating behaviour following the introduction of a sexually receptive female (Fig. 2a). Ca^2+^ signals increased slightly during the chasing phase and peaked immediately after the onset of successful mounting (Fig. 2b and Supplementary Video 1). We observed a strong Ca^2+^ increase from all test mice (Δ*F/F*=47.6%±11.8%, mean±s.e.m., *n*=6 *SERT*-DRN^GCaMP6^ mice; Fig. 2c and Supplementary Fig. 2a). The activation remained significant throughout the mounting episodes (*P*<0.05, permutation test, 9.0±1.3 s for GCaMP signals versus 9.4±1.3 s for mounting, mean±s.e.m., *n*=6 mice). We did not observe any significant change of fluorescence signals from *SERT*-DRN^EmGFP^ control mice (*P*>0.05, permutation test, *n*=4 mice; Supplementary Fig. 2b), again indicating that the mating-associated fluorescence increase in *SERT*-DRN^GCaMP6^ mice was produced by Ca^2+^ changes in 5-HT neurons.

To examine the effect of male–male interaction, we introduced a novel male mouse into the home cage of the test male and recorded GCaMP fluorescence for 5 min. The test male frequently investigated the body of the intruder male (Fig. 2d). The chemoinvestigation were associated with increase of GCaMP fluorescence that peaked when the nose of the test mouse touched the body of the social target (Fig. 2e). We observed significant Ca^2+^ signals from all test mice (Δ*F/F*=23.7±3.4%, mean±s.e.m., *P*<0.05, permutation test, *n*=7 mice; Fig. 2f and Supplementary Fig. 2c). The duration of activation was sublinear to the duration of chemoinvestigation (5.6±1.4 s for GCaMP signals versus 9.5±1.9 s for investigation).

As novelty is rewarding^50^, we tested whether novelty alone underlay the Ca^2+^ signals in social interactions. In separate behavioural sessions, we exposed the test male to a novel inanimate object (a cloth dummy mouse) in the home cage (Fig. 2g). Investigation of the neutral object was associated with small and brief Ca^2+^ transients (Δ*F/F*=17.0±2.1%, 0.5±0.1 s for GCaMP signals versus 0.8±0.2 s for investigation, mean±s.e.m., *n*=7 mice, Fig. 2h,i and Supplementary Fig. 2d). Overall, mating produced substantially stronger Ca^2+^ signals than chemoinvestigation of another male, which in turn produced stronger and much more prolonged responses than object investigation (Supplementary Fig. 2e). The signal amplitudes (peak Δ*F/F*) were poorly correlated with the duration of behavioural episodes (Supplementary Fig. 2f), indicating that behaviour types rather than duration determined Ca^2+^ signal amplitudes. Therefore, social rewards activate DRN 5-HT neurons more effectively than general novelty.

### Unexpected rewards activate 5-HT neurons

**Figure 3:**
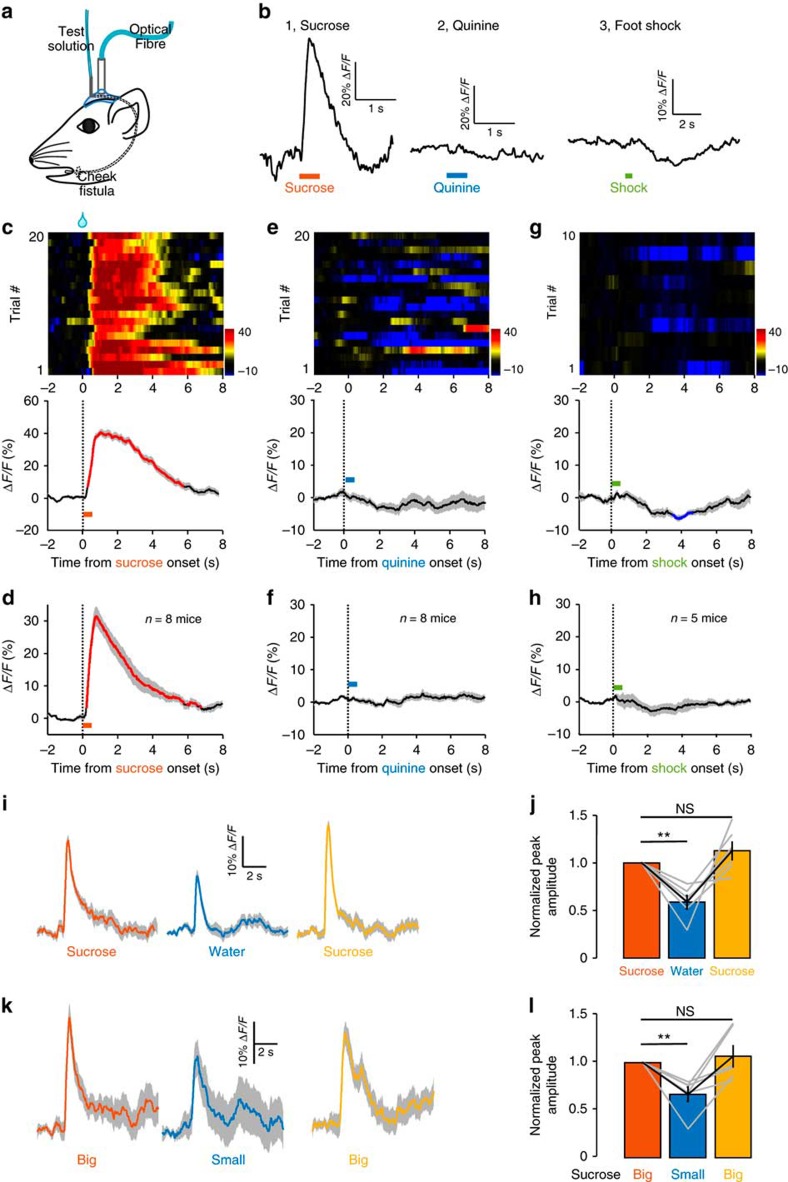
DRN 5-HT neurons are activated by unexpected delivery of sucrose but not aversive stimuli. (**a**) Schematics of recording Ca^2+^ signals in response to intraoral solution delivery through a cheek fistula. (**b**) Representative raw traces of GCaMP fluorescence changes to random delivery of sucrose (b1), quinine (b2) and footshock (b3). Ca^2+^ response patterns of DRN 5-HT neurons from a representative mouse (**c**) and the entire test group (**d**, *n*=8 SERT-DRN^GCaMP6^ mice). Sucrose solution was randomly infused through a cheek fistula for 20 trials in one behavioural session. Ca^2+^ transients were aligned to the onset of 0.5 s infusion. Lines indicate average and the shaded area indicate s.e.m. Red lines represent statistically significant increase from the base line (*P*<0.05; permutation test). GCaMP fluorescence change to intraoral delivery of quinine for one mouse (**e**) and the test group (**f**, *n*=8 SERT-DRN^GCaMP6^ mice). Footshock-associated Ca^2+^ signals from a representative mouse (**g**) and the test group (**h**, *n*=5 SERT-DRN^GCaMP6^ mice). Blue line in **g** indicate significant decrease. (**i**) The GCaMP signals of 30 consecutive trials were averaged for sucrose (5%), pure water and sucrose again (5%). (**j**) Summary of data on the effect of replace sucrose with water. **P<0.01; NS, not significant; multiple comparisons after repeated measures one-way analysis of variance (ANOVA); *n*=6 mice. Error bars indicate s.e.m. (**k**,**l**) Effects of reward size. Same conventions as in **i** and **j**, except that big reward consisted of 8 μl 5% sucrose in 0.5 s and small reward had only half volume (2 μl in 0.5 s). Error bars in **l** indicate s.e.m.

Our results so far demonstrate that DRN 5-HT neurons are activated when a mouse voluntarily seeks rewards and receives them. We next asked what if the mouse received reward unexpectedly—without seeking or effort. We implanted intraoral cheek fistulae to deliver sucrose solution into the oral cavity of a water-deprived mouse (Fig. 3a)^51^. This approach allowed us to precisely control the volume and duration of sucrose delivery. Reward expectation was minimized by dispensing sucrose solution randomly with an inter-trial interval of 20–40 s. Sucrose delivery (5% w/v, 10 μl in 0.5 s) reliably elicited Ca^2+^ transients from 5-HT neurons of a *SERT*-DRN^GCaMP6^ mouse (Fig. 3b1). Significant Ca^2+^ increase was observed from all test mice (Δ*F/F*=40.0±3.4%, mean±s.e.m., *P*<0.05, permutation test, *n*=8 mice; Fig. 3d and Supplementary Fig. 3a). The Ca^2+^ signals rose and decayed rapidly (median latency to peak=0.84 s, decay duratio*n*=3.82±0.65 s, mean±s.e.m.; Fig. 3d and Supplementary Fig. 3b). Therefore, sucrose transiently activates 5-HT neurons even when the mouse is not expecting it.

To test whether 5-HT neurons responded to general saliency rather than rewarding quality of sucrose, we examined the effect of two aversive stimuli, a bitter tastant and footshock. Random intraoral delivery of quinine (5 mM, 10 μl in 0.5 s) reduced Ca^2+^ signals in some trials (Fig. 3b2,e), but the inhibition was not statistically significant for the entire test population (*P*>0.05, *n*=8 mice; permutation test; Fig. 3f and Supplementary Fig. 3c). Similarly, electric footshock resulted in a trend of Ca^2+^ signal reduction that were not statistically significant for the entire test group (*P*>0.05, *n*=5 mice, permutation test; Fig. 3b3 and Supplementary Fig. 3d). Thus, aversive stimuli do not activate the 5-HT neuron population in the DRN, indicating that 5-HT neurons encode reward but not general saliency.

To examine the effect of reward value, we compared the GCaMP signals produced by sucrose (5%) with those by water alone. Within a single session of 90 trials, we applied 30 trials of sucrose (5%, 10 μl in 0.5 s), followed by 30 trials of water (10 μl in 0.5 s) and additional 30 trials of sucrose. GCaMP signals in both sucrose blocks were significantly larger than water-evoked signals (*P*<0.01, Dunn's multiple comparisons test, *n*=6 mice; Fig. 3i,j). To examine the effect of reward size, we varied the volume of sucrose drops by adjusting infusion speed (16 versus 4 μl s^−1^) to generate ‘big' rewards (8 μl in 0.5 s) and ‘small' rewards (2 μl in 0.5 s). Big rewards evoked significantly stronger GCaMP signals than small rewards (*P*<0.01, Dunn's multiple comparisons test, *n*=6 mice; Fig. 3k,l). Thus, reward quality and size modulate the response amplitudes of 5-HT neurons.

### Expected reward activates 5-HT neurons before delivery

**Figure 4:**
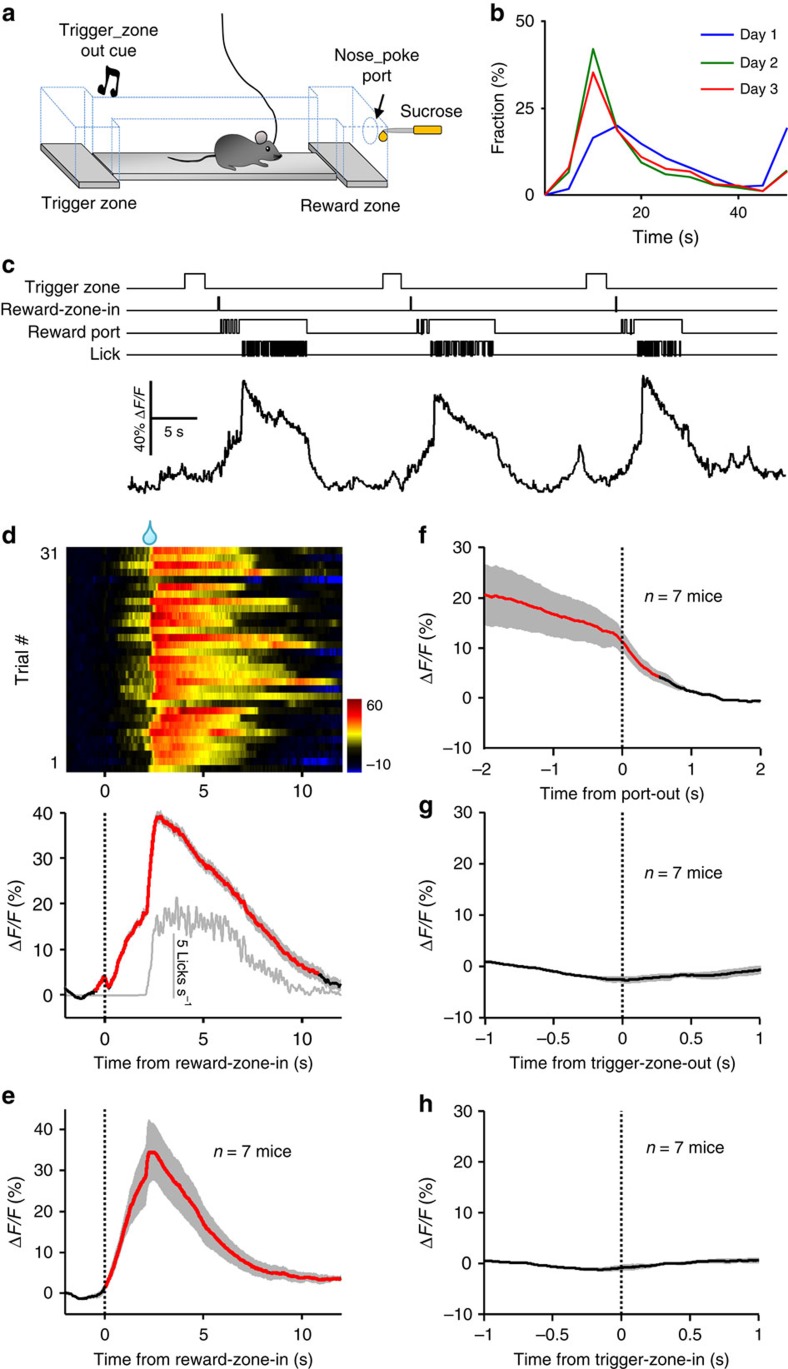
Ca^2+^ transients of DRN 5-HT neurons during a sucrose foraging task. (**a**) Schematics of the behavioural task. A mouse ran from the trigger zone to the reward zone and poked its nose through the reward port for delayed delivery of sucrose. (**b**) Distribution of trial duration between exit from the trigger zone and return from the reward zone for daily behavioural sessions. (**c**) Raw trace of Ca^2+^ transients from a SERT-DRN^GCaMP6^ mouse during the sucrose foraging task. Sucrose was delivered with 2 s delay following reward-zone entry. TTL signals above the Ca^2+^ trace indicate various behavioural events. (**d**) Example heatmap (top) and peri-event plots (bottom) of fluorescence changes aligned to mouse entries to the reward zone (reward-zone-in). Each row in the heatmap draws the data from one behavioural trial and a total of 31 trials are plotted. The grey line in the lower panel indicates lick rates. Thick lines indicate mean and shaded areas indicate s.e.m. Red segment indicates significant increase (*P*<0.05; permutation test). (**e**) Population data on the mean Ca^2+^ transients of DRN 5-HT neurons following reward-zone-in events (*n*=7 SERT-DRN^GCaMP6^ mice). Mean Ca^2+^ signals aligned to the termination of nose pokes through the reward port (**f**), trigger-zone-out events (**g**) and trigger-zone-in events (**h**). *n*=7 SERT-DRN^GCaMP6^ mice.

How do 5-HT neurons respond when a thirsty mouse expects a reward but is made to wait? We addressed this question by training mice with a simplified sucrose foraging paradigm^52^. A water-deprived mouse learned to move back and forth between the chamber of ‘trigger zone' and the chamber of ‘reward zone' to collect sucrose drops (Fig. 4a). Exit from the trigger zone triggered an auditory tone to signal trial initiation. Following entries into the reward zone, a mouse typically started poking into the reward port and licked a nozzle for sucrose, which was supplied through a solenoid valve briefly triggered open (50 ms) with a 2-s delay following reward-zone entry. Initially, a thirsty mouse explored and accidentally received sucrose solution from the liquid nozzle. Following two daily training sessions, a mouse learned to move repeatedly from the trigger zone to the reward zone to acquire sucrose drop following the delay (Fig. 4b and Supplementary Fig. 4a,b). By controlling delay duration and reward outcome, we could precisely correlate the Ca^2+^ signals of 5-HT neurons to various parameters of the self-paced reward-seeking behaviour, such as reward-zone entry, waiting and sucrose acquisition.

In trained mice, the Ca^2+^ signals ramped up when a mouse entered the reward zone, remained elevated throughout the 2-s delay period and further increased when the mouse finally received the sucrose drop (Fig. 4c,d). On entry to the reward zone, the entire test group reached the peak Δ*F/F* of 38.9±2.6% (mean±s.e.m.) after a median latency of 2.3 s (*n*=7 mice; Fig. 4e and Supplementary Fig. 4c–e). The Ca^2+^ signals rapidly decayed to the baseline after sucrose delivery (Fig. 4f), although the mouse continued to lick for a few more seconds (Supplementary Fig. 4d,e). We did not observe any clear change of Ca^2+^ signals correlated to the events of trigger-zone exit and re-entry (Fig. 4g,h). Therefore, 5-HT neuron activation is specifically related to waiting and sucrose acquisition in the reward zone.

**Figure 5:**
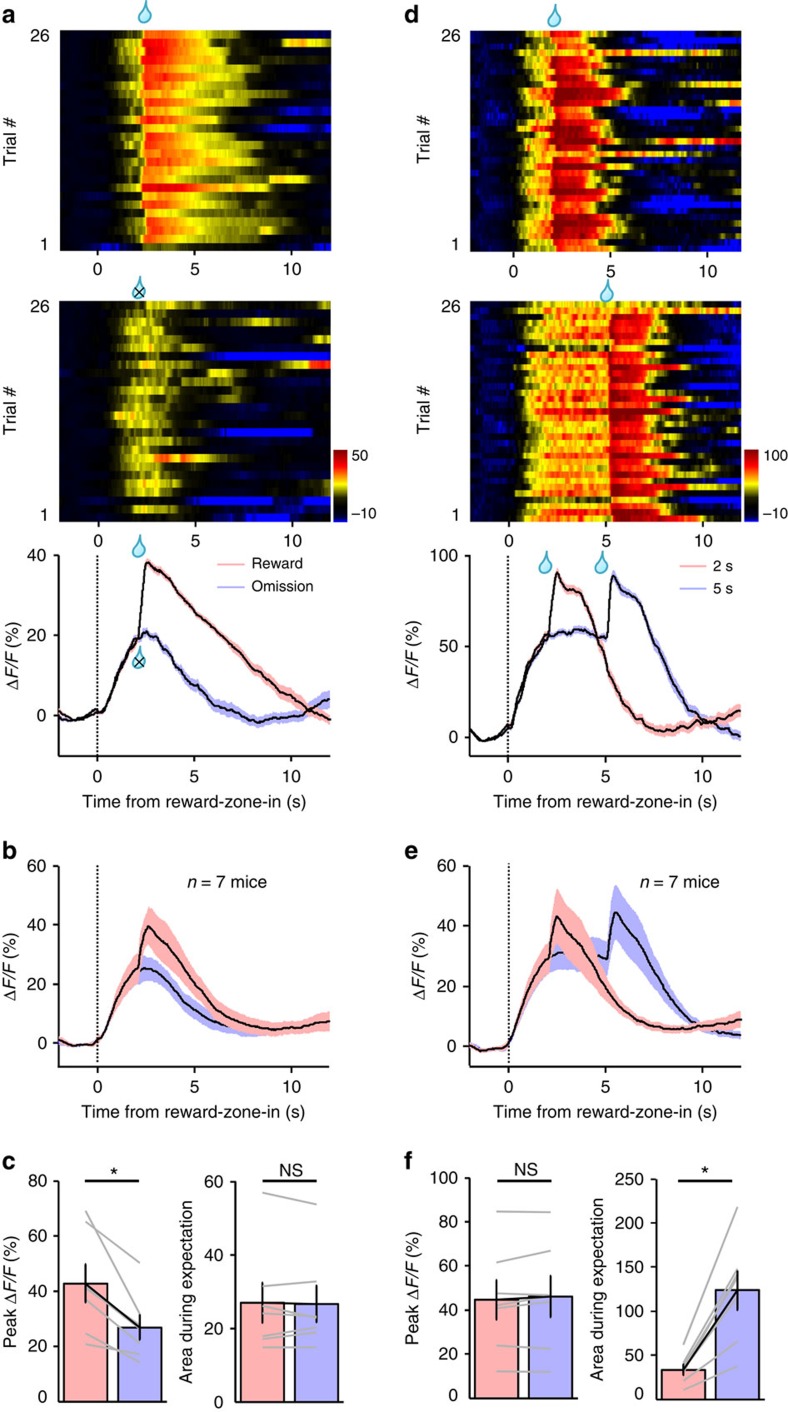
In trained mice, DRN 5-HT neurons are activated during reward waiting and following reward acquisition. (**a**–**c**) The effect of sucrose omission on Ca^2+^ signals. Sucrose solution was randomly omitted for half of trials in a sucrose foraging session. The heatmap in **a** illustrates the GCaMP signals of a representative mouse for sorted trials of sucrose delivery (upper) and sucrose omission (lower). The plots show the average Ca^2+^ signals of the single mouse (**a**) and the entire test group (**b**, *n*=7 mice). Thick lines indicate mean and shaded areas indicate s.e.m. The bar graphs in **c** show that sucrose omission significantly reduced Ca^2+^ signal amplitude associated with scheduled sucrose delivery (left) but did not affect the Ca^2+^ signal during reward delay (right). Lines indicate data from individual mice. *P<0.05; NS, not significant; Wilcoxon's signed-rank tests. *n*=7 mice. (**d**–**f**) The effect of extending the delay length on Ca^2+^ signals. Trials were randomly assigned with the delay of either 2 or 5 s in the same test session. The Ca^2+^ response of a single mouse is shown in **d** and the population response is shown in **e**. *P<0.05; NS, not significant; Wilcoxon's signed-rank tests. *n*=7 mice.

We varied the reward outcome and delays to dissociate their effects on Ca^2+^ signals. In separate behavioural sessions, sucrose drops were either delivered following a 2-s delay or randomly omitted in half of trials. The Ca^2+^ signals were identical during the 2-s delay period for both trials of reward delivery and those of omission (Fig. 5a–c). However, sucrose omission abolished the additional transient increase associated with sucrose delivery (*P*=0.016, Wilcoxon's signed-rank tests, *n*=7 mice; Fig. 5a–c and Supplementary Fig. 4d,e), demonstrating an excitatory effect of reward acquisition on 5-HT neurons.

In separate behavioural sessions, we randomly assigned delays of 2 or 5 s to equal number of trials. Increasing the delay extended the initial plateau of Ca^2+^ signals to 5 s and the signals further increased following sucrose acquisition (*n*=7 mice; Fig. 5d–f). During the delay period, three out of the seven test mice started licking during the waiting period before sucrose delivery, whereas the other four mice licked only after sucrose delivery (Supplementary Fig. 4d). Regardless of the difference in licking behaviour, all mice exhibited strong Ca^2+^ signals during the delay (Supplementary Fig. 4e), indicating that waiting-associated activation does not depend on lick movement.

### 5-HT neurons exhibit the tonic-then-phasic activation pattern

**Figure 6:**
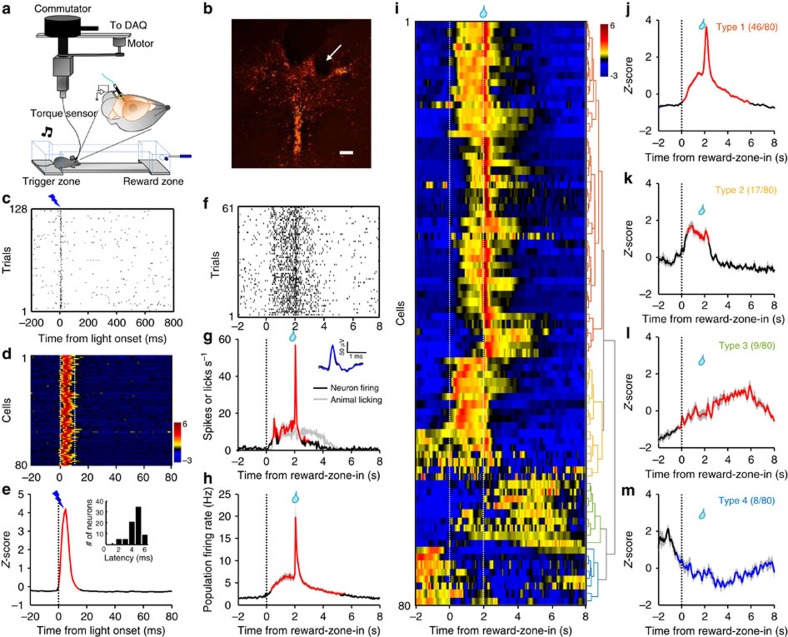
Spike firing pattern of individual 5-HT neurons during the foraging task. (**a**) The method of optetrode recording from DRN 5-HT neurons of freely behaving mice. Torque signals from the recording cable controlled a motorized commutator to ease mouse movement. (**b**) The location of electrolytic lesion, pointing to a recording site within the DRN of a SERT-DRN^ChR2^ mouse. Red, ChR2-mCherry. Scale bar, 200 μm. (**c**–**e**) The method of optogenetic tagging. The raster plot in **c** shows the effect of single light pulses (5 ms) on evoking spike firing from a single cell. Each line indicates a 1-s trial. Heatmap in **d** shows the *Z*-score representation of light-evoked spiking activity for all identified 5-HT neurons (*n*=80 cells). Each line plots the response of one cell. Dash lines delineate 10-ms interval following light onset. (**e**) Average *Z*-scores of spike firing rates (*n*=80 cells). Inset in **e** plots the distribution histogram of spike latency. (**f**,**g**) Spike firing activity of one representative 5-HT neuron. The raster plot in **f** is derived from the firing activity across trials for the same cell shown in **c**. Thick lines indicate mean and shaded areas indicate s.e.m. The PETH (smoothed with a Gaussian kernel with σ of 50 ms) of spike firing rates (red) and lick rates (grey) are aligned to the reward-zone-in event (**g**). Inset illustrates the waveform overlay of average spontaneous spikes (black) and average light-evoked spikes (blue). (**h**) Mean PETH of spike firing rates of all identified 5-HT neurons (*n*=80 cells). (**i**) The activity patterns of individual 5-HT neurons. The PETHs of *Z*-scored spike firing rates are represented in the heatmap format (colorscale at the top right). Each row indicates the activity pattern of one cell aligned to the reward-zone-in event (the left dash line). The right dash line indicates sucrose delivery. Data were hierarchically clustered based on the first three major principal components of *Z*-scores (dendrogram at the right). Cluster colours denote the four major response subtypes based on the cluster analysis. (**j**–**m**) Mean *Z*-scores of the four subtypes of 5-HT neurons. Red indicates significant increase and blue indicates significant decrease from the baseline (*P*<0.05; permutation test). Shaded areas indicate s.e.m.

To examine the electrophysiological basis of Ca^2+^ signals and the response pattern of individual neurons, we carried out single-unit recordings from the DRN, while a mouse performed the sucrose foraging task (Fig. 6a). Extracellular spikes were recorded with a microdrive-controlled optetrode consisting of four tetrodes and a single optical fibre^43^,^53^. Torque signals from the recording cable actively controlled a motorized commutator to facilitate mouse moving^54^. For *in vivo* optogenetic tagging of 5-HT neurons, we expressed Channelrhodopsin-2 (ChR2) by infusing AAV-DIO-ChR2-mCherry vectors into the DRN of *SERT-Cre* mice (Fig. 6b). After isolation of single units (Supplementary Fig. 5a), we examined whether brief light pulses (5 ms) could reliably and rapidly elicit spike firing (Fig. 6c–e). We referred to the statistical method that was recently developed for analysing the significance of tagging^4^,^52^.

We recorded 80 optogenetically identified 5-HT neurons from 14 mice. Most of 5-HT neurons exhibited activation in the reward zone (‘reward-on') (Fig. 6f,g). The spike firing rates of a 5-HT cell were often low outside the reward zone (<1 spike s^−1^) and increased to ∼10 spikes s^−1^ within the reward zone. The neuronal activity remained elevated, while the mouse waited, briefly soared to as high as 60 spikes s^−1^ following sucrose delivery and then decreased to baseline in 2 s (Fig. 6f,g). Population average revealed that in general, 5-HT neurons fired tonically at ∼6 spikes s^−1^ during waiting and then phasically at the peak rate of ∼20 spikes s^−1^ on sucrose delivery (tonic-then-phasic pattern; Fig. 6h). There was no activation when a mouse entered or left the trigger zone (Supplementary Fig. 5b,c), indicating that neuronal activation were specific to the behaviour events in the reward zone.

Some 5-HT neurons deviated from the typical tonic-then-phasic pattern (Fig. 6i). Principal component analysis and hierarchical clustering classified 5-HT neurons into four subtypes. In addition to the typical type-1 cells (46/80 cells, 58%; Fig. 6i,j), type-2 cells (17/80 cells, 21%) exhibited low firing activity in the trigger zone, tonic activation during waiting in the reward zone and weak or no phasic response following sucrose delivery (Fig. 6i,k and Supplementary Fig. 5d,e). Type-3 cells (9/80 cells, 11%) were more active in the trigger zone and exhibited mild but sustained excitation following reward zone entry (Fig. 6i,l and Supplementary Fig. 5d,f). Finally, type-4 5-HT cells (8/80 cells, 10%) also showed higher spontaneous activity but were inhibited for up to 10 s following reward zone entry (‘reward-off'; Fig. 6i,m and Supplementary Fig. 5d,g).

Compared with the optogenetically tagged 5-HT neurons, non-tagged DRN neurons responded much more heterogeneously (*n*=129 cells; Supplementary Fig. 6). For example, the percentage of ‘reward-off' cells was substantially higher (35% non-tagged neurons versus 10% tagged 5-HT neurons), demonstrating the importance of genetic identification for accurately revealing the response properties of 5-HT neurons.

Given that reward delay and outcome affected the Ca^2+^ signals of 5-HT neuron populations (Fig. 5), we examined the effects of omitting rewards and extending delay on the spike firing of single units. Regardless of reward outcome, 5-HT neurons exhibited similar tonic activation when mice waited during the 2-s delay, but sucrose omission eliminated the burst firing associated with sucrose delivery (*n*=20 cells; Supplementary Fig. 7a,b). Increasing the reward delay from 2 to 5 s extended the tonic activation to 5 s, which was switched to brief bursting following reward delivery (*n*=22 cells; Supplementary Fig. 7c,d). Thus, the tonic excitation and phasic firing of 5-HT neurons correspond to waiting and reward acquisition, respectively.

### DRN GABA neurons are inhibited during reward seeking

We performed fibre photometry to investigate how DRN GABA neurons respond in reward-associated tasks. We expressed GCaMP6 in GABA neurons by injecting AAV-DIO-GCaMP6m into the DRN of *VGAT-Cre* mice (Supplementary Fig. 8a), in which Cre expression was directed to GABA neurons by the promoter region of the gene encoding vesicular GABA transporter (VGAT)^55^. We tested the effects of sucrose lick, food intake, intraoral delivery of sucrose and quinine, and footshock on GCaMP6 fluorescence in eight VGAT-DRN^GCaMP6^ mice.

**Figure 7:**
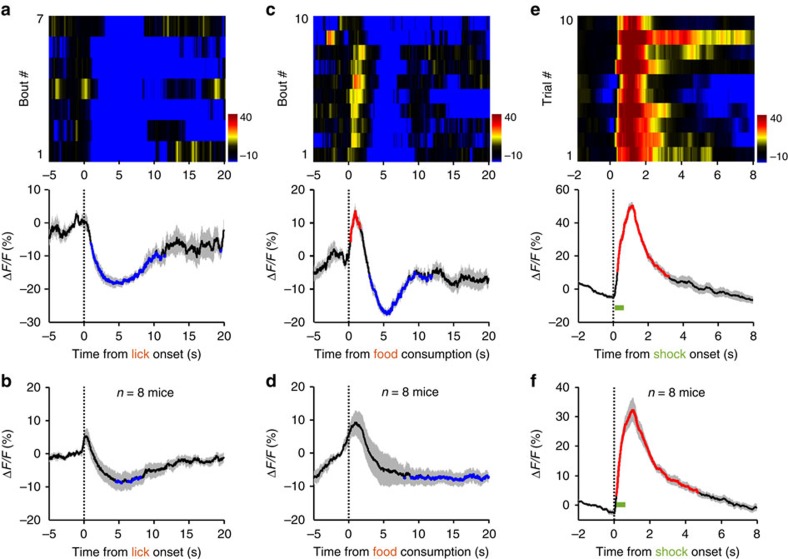
The effects of appetitive and aversive stimuli on the Ca^2+^ signals of DRN GABA neurons. Results from a VGAT-DRN^GCaMP6m^ mouse (**a**) and the population data (**b**; *n*=8 mice) showing Ca^2+^ signals associated with sucrose lick. Each row in the upper panel of **a** represents one licking bout in a behaviour session. Thick lines indicate mean and shaded areas indicate s.e.m. Red and blue line segments in the plots represent significant increase and decrease of Ca^2+^ signals (*P*<0.05; permutation tests), respectively. The effect of food intake on the Ca^2+^ signals of DRN GABA neurons from a single mouse (**c**) and the entire test population (**d**; *n*=8 mice). The effect of random footshocks on Ca^2+^ signals in DRN GABA neurons from a single mouse (**e**) and the entire test population (**f**; *n*=8 mice).

The Ca^2+^ signals of DRN GABA neurons typically decreased when a mouse voluntarily licked for sucrose (Fig. 7a). Seven out of eight test mice showed this inhibitory response and the inhibition was significant for the entire test population (*P*<0.05; permutation test; *n*=7 mice; range of Δ*F/F*=(−5 to −20%); duration of inhibition=8.4±1.1 s versus duration of lick=13.9±0.9 s; mean±s.e.m.; Fig. 7b and Supplementary Fig. 8b). Food intake resulted in more variable responses. Six of eight test mice exhibited an initial brief activation (∼1 s) followed by more prolonged inhibition, whereas the other two mice showed slow activation (Fig. 7c and Supplementary Fig. 8c). On average, food intake was associated with a mild but significant reduction of Ca^2+^ signals (*P*<0.05; permutation test; *n*=8 mice; duration of inhibition=10.4±2.0 s; mean±s.e.m.; Fig. 7d). The inhibition probably requires voluntary seeking, because random intraoral delivery of sucrose did not change Ca^2+^ signals (*n*=6 mice; Supplementary Fig. 8d,e).

We tested the effect of two aversive stimuli, quinine and footshock, on the activity of GABA neurons. Intraoral delivery of quinine did not affect Ca^2+^ signals (Supplementary Fig. 8f,g). However, footshock reliably elicited rapid and strong increase of Ca^2+^ signals. The Ca^2+^ transients reached the peak within 1 s of footshock onset and decayed to baseline in 3 s (Δ*F/F*=44.6±3.8%; median latency to peak=1.0 s; decay duratio*n*=2.8±0.5 s; mean±s.e.m.; *n*=8 mice; Fig. 7e,f and Supplementary Fig. 8h,i). Thus, aversive stimuli activate GABA neurons in a modality-dependent manner.

We carried out single-unit recordings of DRN GABA neurons from *VGAT-ChR2-EYFP* transgenic mice (Supplementary Fig. 9a,b)^56^. Optical tagging identified 45 GABA neurons from four *VGAT-ChR2-EYFP* mice that underwent training for the sucrose foraging task (Supplementary Fig. 9c). Outside the reward zone, GABA neurons fired spikes at over ten times higher frequency than 5-HT neurons (18.2±2.2 versus1.6±0.2 spikes s^−1^; mean±s.e.m.; *P*<0.001; Wilcoxon's rank-sum test; *n*=45 GABA cells and 80 5-HT cells; Supplementary Fig. 9d).

**Figure 8:**
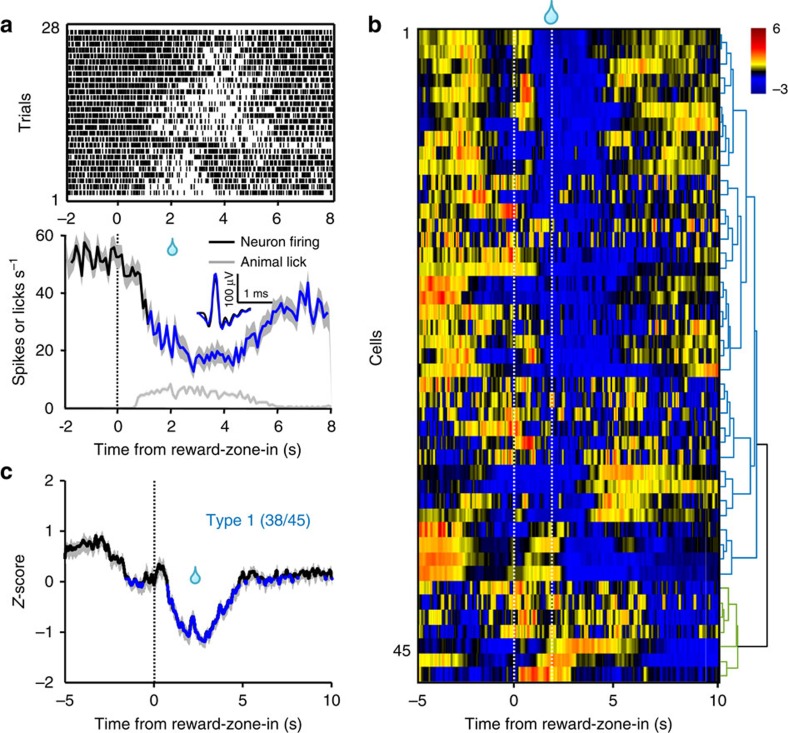
The spike firing activity pattern of individual DRN GABA neurons during the foraging task. (**a**) The activity pattern of a DRN GABA neuron from a VGAT-ChR2-EYFP mouse. Upper panel, spike firing rate aligned to the reward-zone-in events of each trial. Lower panel, the plots of neuronal firing rates and mouse lick rates across trials. Inset shows the waveform overlay of average spontaneous spikes (black) and average light-evoked spikes (blue). Thick lines indicate mean and shaded areas indicate s.e.m. Blue line segments in the plots represent significant decrease of Ca^2+^ signals (*P*<0.05; permutation tests). (**b**) *Z*-score representation of the activity patterns of individual GABA neurons (*n*=45 cells). Each row indicates one cell. Data were hierarchically clustered. The two dashed lines indicate reward-zone-in events and sucrose delivery. (**c**) Mean PETH of *Z*-scores for the 38 type-1 GABA neurons.

Most GABA neurons reduced spike firing when a mouse sought sucrose in the reward zone. After a mouse entered the reward zone, a GABA cell decreased its spike firing rates gradually to ∼10 spikes s^−1^ at the point of reward delivery, remained low while the mouse continued to lick and then recovered when the mouse ceased to poke its nose into the reward port (Fig. 8a and Supplementary Fig. 9e,f). Cluster analysis revealed that 38 cells (84%) exhibited this typical ‘reward-off' activity pattern (type-1 GABA cells; Fig. 8b,c). In addition to the typical type-1 GABA cells, a small number of GABA cells (type-2 GABA cells, 7/45 cells, 16%) did not show significant activity change that was associated with behavioural events in the reward zone or the trigger zone (Supplementary Fig. 9e–i). Despite the presence of this small subset of nonresponsive cells, on average the entire population of GABA cells suppressed their activity when the animals waited and acquired rewards (Supplementary Fig. 9j).

## Discussion

We studied how reward signals are encoded by DRN 5-HT and GABA neurons using fibre photometry and single-unit recordings from freely behaving mice. The validity of fibre photometry was demonstrated by the similarity between Ca^2+^ and electrophysiological signals in the sucrose foraging task. We find that 5-HT neurons are activated by diverse natural rewards, including food and social rewards. Moreover, a majority of 5-HT neurons encodes reward expectation and acquisition using the tonic-then-phasic firing pattern. Finally, GABA neurons are inhibited during reward seeking.

One key finding is that genetically identified 5-HT neurons are activated by different types of natural rewards. Strong Ca^2+^ transients were evoked by sucrose, food, sex and social interaction, but not by the aversive stimuli of quinine and footshock. DRN 5-HT neurons thus encode not only liquid-based reward signals but also food and social rewards. As sucrose lick, food intake, mounting a female and chemoinvestigation of another male engage different motor acts, the reward responses are unlikely linked to specific motor behaviour^27^. The lack of Ca^2+^ responses to quinine and footshock indicates that the rewarding quality but not general saliency mediates neuronal activation. For all rewards tested, the Ca^2+^ signals rise and decay in seconds. Therefore, in addition to slowly modulating mood at the time scale of minutes to hours^15^, DRN 5-HT neurons can rapidly broadcast reward signals from different modalities to organize a broad array of reward-related behaviours including drinking, feeding, social interaction, emotion and cognition^13^.

Moreover, DRN 5-HT neurons are activated when an animal receives rewards that are either expected or surprising. Ca^2+^ signals increase when a mouse voluntarily seeks and receives a reward. More importantly, random intraoral delivery of sucrose produces similarly strong activation, thus excluding the potential contribution of reward expectation. After a mouse learns to wait for sucrose delivery in the foraging task, earning the sucrose results in additional Ca^2+^ increase and phasic firing of spikes, indicating that acquisition of expected rewards also activates 5-HT neurons. These observations are in line with the prior reports that the activity of some DRN neurons in primates tracks the actual reward values of water or juice^29^. Through genetic tagging, this study demonstrates that the reward-tracking cells are serotonergic, suggesting that 5-HT neurons and the 5-HT signalling system mediate reward consummatory behaviours.

To obtain reward following a predicting sensory cue, an animal needs to analyse the cue, approach, wait, consume and finally evaluate reward value and cost. When a mouse waits for sucrose, both Ca^2+^ signals and spike firing rates are increased during the entire delay period and further enhanced following sucrose delivery. Possibly because of higher temporal precision of electrophysiological recordings, 5-HT neurons exhibit phasic firings of action potentials upon reward delivery. Manipulating delays and reward outcome further revealed that the initial tonic activation corresponds to reward waiting and the later phasic firing corresponds to reward acquisition. Several earlier recordings reported that a subset of DRN neurons were activated when animals waited for reward^20^,^57^, again without strong evidences that they were indeed 5-HT cells. The tonic-then-phasic activation pattern of optogenetically verified 5-HT neurons may reconcile recent debates on whether 5-HT neurons function before or after reward acquisition^17^,^21^,^22^,^58^,^59^,^60^. Using different activation modes, these neurons may perform the dual roles of expecting and evaluating rewards.

There exists some heterogeneity among the response patterns of individual 5-HT neurons. Although the tonic-then-phasic mode was typical, some cells are activated only during the delay and some exhibit mild but prolonged activation following reward acquisition. A small number of 5-HT neurons (10%) are even inhibited during waiting and reward acquisition (Fig. 3). 5-HT neurons exhibit different neurochemical phenotypes^36^,^61^,^62^,^63^. For example, a substantial number of 5-HT neurons express VGLUT3 and are probably glutamatergic, whereas some 5-HT neurons seem to be purely serotonergic and even smaller percentage of 5-HT neurons expresses GABA markers^17^,^34^,^36^,^64^,^65^. Genetic manipulation that combine multiple markers may help determine whether the response subtypes correspond to neurochemical phenotypes.

Our experiments show a very different picture from what was described by two recent recordings from head-fixed mice^17^,^39^. Liu *et al*.^17^ found that ∼60% of Pet-1 neurons were tonically activated following reward-predicting cue. Possibly because the *ePet1-Cre* mice were head restraint and trained with classical conditioning, and because some Pet-1 neurons (up to 10%) lacked the markers for 5-HT neurons, Pet-1 neurons showed higher spontaneous firing rates (5 versus 1 spikes s^−1^ here) and lacked phasic responses to sucrose delivery^17^. Cohen *et al*.^39^ reported that, out of 29 optogenetically tagged SERT neurons, 22 cells were robustly and phasically activated by the aversive stimulus of air puff and only 11 cells were weakly and slowly activated by unexpected water delivery (typically <3 spikes s^−1^). Immediately following reward-predicting cues, 15 cells were briefly (duration ∼200 ms) activated and 2 were briefly inhibited^39^. From this study, it appeared that 5-HT neurons respond more strongly to aversive stimuli than to rewards^39^. In contrast, we find that 5-HT neurons are activated predominantly by rewards but not aversive stimuli. During reward waiting, 5-HT neurons exhibit continuous activation throughout the tested delay of up to 5 s. This activation pattern matches well with the theory that 5-HT neurons promotes patience during reward waiting^21^,^22^,^59^,^60^. Key technical differences may cause the obvious discrepancies. We recorded from freely behaving mice that are probably much less stressed than head-fixed mice undergoing chronic, inescapable restraint. In addition, we used 5% sucrose solution, which is more rewarding than water alone^17^.

Some 5-HT neurons may also be involved in processing aversive signals^66^,^67^. In the mouse DRN, air puff evoked brief action potential firing from a majority of 5-HT neurons^39^. In the monkey DRN, air puff slowly and mildly modulated the activity of reward-responsive neurons^31^. Here, the aversive stimuli of quinine and footshock do not evoke any obvious Ca^2+^ transients from 5-HT neurons, although footshock reliably activates GABA cells. It cannot be excluded that aversive stimuli do activate some 5-HT cells, but the activation may be too weak and/or brief to produce detectable Ca^2+^ signals. The type-4 5-HT neurons may be the candidate subset of neurons that positively encode aversive signals, as they are inhibited by reward.

In contrast to 5-HT neurons, GABA cells are mostly inhibited by reward. Voluntary sucrose lick and food intake reduce Ca^2+^ signals in GABA cells, indicating inhibition from their high baseline activity. Consistently, most of individual GABA cells show prolonged inhibition when a mouse waits and receives reward in the sucrose-foraging tasks. On the other hand, these cells are strongly activated by footshock, suggesting excitation by painful stimuli. GABA cells receive overlapping but different inputs from those to 5-HT neurons, interact with local 5-HT neurons, and may even project axons outside the DRN^6^,^8^,^40^,^41^. Optogenetic inhibition of GABA cells disinhibits 5-HT cells and prevents the acquisition of social avoidance in mice exposed to social threats^40^. Suppressing the activity of GABA cell may facilitate the activation of DRN 5-HT neurons and contribute to reward signalling. Thus, 5-HT neurons and GABA neurons may have complementary reward codes and synergistically organize behavioural responses to rewards and aversive stimuli.

## Methods

### Mice

Animal care and use conformed to institutional guidelines of the National Institute of Biological Sciences, Beijing, and to the governmental regulations of China. We used a total of 41 (39 males, 2 females; 8–14 weeks old) *SERT-Cre* mice (strain name B6.Cg-Tg(Slc6a4-Cre)ET33Gsat; MMRRC, Davis, CA, USA^48^), 8 (all males, 8–14 weeks old) *VGAT-Cre* mice (strain name B6.FVB-Tg(Slc32a1-cre)2.1Hzo/FrkJ; Jackson Laboratory, Ben Harbor, Maine, USA) and 4 (all males, 8–14 weeks old) *VGAT-ChR2-EYFP* mice (Jax strain name B6.Cg-Tg(Slc32a1-COP4*H134R/EYFP)8Gfng/J; a gift from G. Feng at MIT^56^). Mice were maintained on a 12/12 light/dark cycle and housed in groups of five for 6–8 weeks. After surgery, mice were housed individually on a reverse light–dark cycle (light off at 0800, h) for at least 1 week before further experiments.

### Surgery and virus injection

Mice were anaesthetized with pentobarbital (intraperitoneal, 80 mg kg^−1^) and mounted to a stereotaxic apparatus equipped with an electric heating pad. The skin was cut and a small craniotomy was made 5 mm posterior to the bregma along the midline. Using a microsyringe pump (Nanoliter 2000 Injector, WPI), AAV virus (500 nl) was slowly injected (40 nl min^−1^) into the DRN through a glass pipette with a 15° angle from caudal to rostral (dorsal–ventral depth=2.5 mm). The glass pipette was left in place for five additional minutes and then slowly withdrawn.

AAV vectors carrying the DIO-ChR2-mCherry, DIO-EmGFP or DIO-GCaMP6m construct were packaged into AAV2/9 serotype with titres 1–5 × 10^12^ viral particles per ml. We constructed these plasmids by replacing the coding region of ChR2-mCherry in the pAAV-EF1α-DIO-hChR2(H134R)-mCherry plasmid (a gift from K. Deisseroth) with those encoding enhanced membrane GFP (Addgene Plasmid 14757) or GCaMP6m (Addgene Plasmid 40754), respectively.

We implanted intraoral cheek fistula following a previously described procedure^51^. The surgery was performed 4 days after the injection of AAV-DIO-GCaMP6m virus into the DRN. An incision was made in the cheek lateral and rostral to the first molar and another incision was made in the scalp in front of the ear or in the back neck. A soft Silastic tubing (30 mm in length, 0.30 mm inner diameter (I.D.) and 0.46 mm outer diameter (O.D.); Dow Corning) was inserted 2 mm into the oral cavity through the cheek incision site and then guided subcutaneously out through the scalp incision. An L-shaped 26-gauge (O.D. 0.48 mm) stainless steel tubing was connected to the Silastic tubing and imbedded aside the ceramic ferrule for optical fibre. A piece of polyethylene tubing (10 mm in length, 0.4 mm I.D. and 1.1 mm O.D.) was fit to the exposed end of the L-shaped tubing. A plug was inserted to the exposed end of the polyethylene tubing, to prevent blockage.

### Fibre photometry

Following AAV-DIO-GCaMP6m virus injection, an optical fibre (230 μm O.D., 0.37 numerical aperture (NA); Shanghai Fiblaser) was placed in a ceramic ferrule and inserted towards the DRN through the craniotomy. The ceramic ferrule was supported with a skull-penetrating M1 screw and dental acrylic. Mice were individually housed for at least 1 week to recover.

To record fluorescence signals, laser beam from a 488-nm laser (OBIS 488LS; Coherent) was reflected by a dichroic mirror (MD498; Thorlabs), focused by a × 10 objective lens (NA=0.3; Olympus) and then coupled to an optical commutator (Doric Lenses). An optical fibre (230 μm O.D., NA=0.37, 2-m long) guided the light between the commutator and the implanted optical fibre. The laser power was adjusted at the tip of optical fibre to the low level of 0.01–0.02 mW, to minimize bleaching. The GCaMP fluorescence was bandpass filtered (MF525-39, Thorlabs) and collected by a photomultiplier tube (R3896, Hamamatsu). An amplifier (C7319, Hamamatsu) was used to convert the photomultiplier tube current output to voltage signals, which was further filtered through a low-pass filter (40 Hz cut-off; Brownlee 440). The analogue voltage signals were digitalized at 500 Hz and recorded by a Power 1401 digitizer and Spike2 software (CED, Cambridge, UK).

### Behavioural tasks

*Sucrose lick and food consumption*. A mouse was water and food deprived for 12 h before it was placed in a chamber (20 × 20 × 22, L × W × H in cm) equipped with a contact lickometer connected to a bottle filled with 5% sucrose solution (w/v). Dustless precision food pellets (14 mg, Bio-serv) were manually delivered to a small platform (1.2 mm diameter, 1 cm height) in the centre chamber floor with only one pellet per time. A Power 1401 digitizer simultaneously recorded the lick signals from the lickometer and fluorescence signals from the photomultiplier tube. Videos (15–20 min per session) from an overhead infrared camera were synchronized with Power 1401 data acquisition. Bout initiation time for food intake was determined by video scoring.

*Intraoral infusion*. A mouse was water deprived for 12 h before sucrose but not quinine infusions. A peristaltic pump (AniLab) delivered either sucrose solution (5% w/v), water or quinine solution (5 mM) through the L-shaped tubing. The pump was controlled through an IC board (Arduino Uno R3) using a self-developed MATLAB programme. To examine the effect of sucrose or quinine, 20 infusions of sucrose or quinine solution (speed 20 μl s^−1^ and duration 0.5 s) were given within one recording session with random intervals ranging from 20 to 40 s. To examine the effect of reward quality, a mouse was tested with 30 consecutive trials of 5% sucrose (speed 20 μl s^−1^ and duration 0.5 s) that were followed by 30 trials of water and then 30 trials of 5% sucrose again. To examine the effect of reward size, a mouse was tested with 30 trials of big reward (5% sucrose, speed 16 μl s^−1^ and duration 0.5 s), 30 trials of small reward (5% sucrose, speed 4 μl s^−1^ and duration 0.5 s) and 30 trials of big reward again. The fluorescence data were aligned to the events of infusion onset.

*Footshock*. Animals were introduced into an acrylic box (25 × 25 × 30, L × W × H in cm) with metal grid floor. In a single test session, ten footshocks (0.7 mA scrambled, 0.5 s; Beijing TianMingHongYuan) were randomly delivered with inter-trial intervals of 20–40 s. Shock delivery onset was used as the trigger event for data alignment.

*Social interaction*. The test male mice were housed individually at least for 1 week after surgery. To study the effect of sexual behaviour, a test mouse was habituated in the dark with an optical fibre connected to the fibre ferrule on its head. A female mouse was introduced into the homecage of the test male so that the male gained sexual experience. A single recording session lasted 30 min and the behaviour of the test mouse was video taped with an overhead infrared camera. Mounting onset was characterized as the placement of the forequarter of the male over the hindquarter of the female. Only mountings with rhythmic contractions of the hindquarter of the male were considered successful mounting.

In the male–male interaction sessions, a test male mouse was placed in its own homecage and the mouse behaviour was video recorded in the dark with an overhead infrared camera. After 1 min habituation, we introduced a group-housed male mouse (intruder) and videoed the social interactions for 5 min. In the separate sessions of object investigation, a cloth-made dummy mouse was introduced in the middle of the cage after 1 min habituation and the animal behaviour was recorded for another 5 min. Offline video scoring identified the events of male–male interaction or object investigation, which was defined as when the test mouse touched the intruder mouse or the object with its nose.

*Foraging*. The simplified foraging task was described in details elsewhere^52^. Briefly, a mouse was kept on a water restriction schedule to maintain 85–90% of free-drinking body weight. The foraging field consisted of a linear track (45 × 5 × 10, L × W × H in cm) that connected with two end chambers (10 × 10 × 8.3 cm; defined as ‘trigger zone' and the ‘reward zone'). The mouse was trained to run from the trigger zone to the reward zone, to collect drops of sucrose solutions (5%, w/v) and to return from the reward zone to the trigger zone before a new trial was initiated. Mouse body positions were monitored using an overhead camera (Logitech C510, 20 FPS). Exit from the trigger zone activated a brief auditory cue (200 ms at 4 KHz square wave with 0.5 duty) to signal the exit from the trigger zone (trigger-zone-out) and thus trial initiation. Reward delays (2 or 5 s) or reward outcomes were set in pseudorandom orders. Sucrose solution was delivered through a metal tubing (1.5 mm O.D., 3 cm above the floor) in the end wall of reward zone and the delivery was controlled with a solenoid valve (20 μl in 50 ms). Two separate infrared photodiodes detected nose poking through the reward port and licking behaviour. Experimental control and behavioural data acquisition were implemented with an IC board (Arduino Uno R3) and a customized MATLAB programme.

Trial start cue was identical in the omission and delay sessions. In omission sessions, omission trials were randomly intermingled with rewarding trials of 2 s delay after animal's entry into the reward zone. The omission was accompanied by the opening of an adjacent solenoid valve at the same delay to minimize potential difference in auditory cues^25^. In delay sessions, sucrose was randomly delivered either at 2 s or at 5 s after reward-zone-in events. A mouse completed 40–80 trials in a single session.

### Electrophysiological recording and optical tagging

To record single units using optical tagging, we adopted the recent design of optetrode microdrives^43^. The microdrive was positioned just above the craniotomy with a protruding optetrode in alignment with the track of glass micropipette for virus injection. The optetrode comprised four tetrodes and one optical ferrule (125 μm diameter, NA=0.37). Tetrodes were twined from 12.7 μm Ni-Cr-Fe wires (Stablohm 675, California Fine Wire, CA, USA) and gold plated to reduce impedance to 250–500 KΩ (ref. 17). The optical fibre was housed in a ceramic fibre optic ferrule (2.5 mm O.D. and 126 μm I.D.). The optic fibre and the tetrodes were then glued together, with the tetrode tips extending 500 μm away from the tip of optical fibre^53^. The optetrode was gradually lowered to a depth of 0.7 mm above the DRN. A silver wire (127 μm dia, A-M System) was attached to one of the four skull-penetrating M1 screws to serve as ground. The microdrive was secured to the skull with dental acrylic. Mice were individually housed for at least 2 weeks for recovery and AAV expression. Food and water were available *ad libitum* until mice started behavioural training using the sucrose foraging paradigm.

A self-developed 16-channel amplifier recorded electrophysiological signals with built-in band pass filters (0.5–3.6 kHz). One of the channels was selected as a virtual reference to minimize moving artefact. Analogue signals were digitalized at 25 kHz and collected using a Power1401 digitizer and Spike2 software. Single units were sorted offline with Spike2 software. A 25-channel commutator (Crist Instruments) was pulled by a torque-controlled servomotor (Faulhaber 2036U), to facilitate torqueless movement of the test mouse^54^. At the end of each recording session, the optetrode was lowered 20–60 μm by manually turning an M1 machine screw in the microdrive. The recording was resumed on the next day unless the electrode tips were judged to be outside the DRN.

Optical tagging confirmed the cell type of recorded single units. Light pulses (5 ms, 0.1 or 10 Hz) were passed through the optical fibre to stimulate action potential firing. The light intensity was lowered to reduce spike jittery. Four parameters were calculated: the reliability of light-evoked spiking within 10 ms from light onset (R), correlation coefficient of spike waveform for spontaneous spikes and evoked spikes (C), the latency of triggering spikes after light onset (L) and a statistical *P*-value to determine whether the spikes were truly evoked by light stimulation^52^. The *P*-value was determined by comparing the distribution latencies of light-evoked spikes and a bootstrapped distribution of latencies of spontaneous spikes^52^. Only neurons with *P*≤0.001 and C≥0.85 were considered as optically tagged neurons^4^,^52^.

### Immunostaining

A mouse was deeply anaesthetized with an overdose of pentobarbital and then intracardially perfused with 0.9% saline followed by 4% paraformaldehyde in PBS. After cryoprotection with 30% sucrose, the mouse brain was sectioned coronally (35 μm thick) with a cryostat (Leica CM1900). For immunohistochemistry, the sections were blocked with 3% BSA in PBS with 0.3% Triton X-100 and incubated with a rabbit polyclonal antibody against Tph2 (1:400, Millipore; 20 h) and chicken polyclonal antibody to GFP (1:400, Abcam) at 4 °C overnight. After washing with PBS, the sections were incubated with Cy3-conjugated goat anti-rabbit IgG (1:500; Jackson ImmunoResearch) and fluorescein-conjugated donkey anti-chicken IgG (1:500; Jackson ImmunoResearch) for 1 h at room temperature. PBS-washed sections were then coverslipped with 50% glycerol mounting medium.

### Slice recording

VGAT-ChR2 mice were deeply anaesthetized with pentobarbital (100 mg kg^−1^ intraperitoneally) and intracardially perfused with ∼5 ml ice-cold oxygenated perfusion solution. The mouse brain was quickly placed in ice-cold oxygenated slicing solution. Coronal sections (250 or 300 μm thick) were prepared with a Leica VT1200S vibratome. The slices were incubated for 1 h at 34 °C with Ringer solution saturated with mixed 95% O_2_ with 5% CO_2_. After incubation, a slice was transferred to a chamber and superfused with ringer solution (2 ml min^−1^) at 28 °C for patch clamp recording. Perfusion solution consisted of (in mM) 225 sucrose, 119 NaCl, 2.5 KCl, 0.1 CaCl_2_, 4.9 MgCl_2_, 1.0 NaH_2_PO_4_, 26.2 NaHCO_3_, 1.25 glucose, 3 kynurenic acid and 1 Na L-ascorbate. Slicing solution consisted of (in mM) 110 choline chloride, 2.5 KCl, 0.5 CaCl_2_, 7 MgCl_2_, 1.3 NaH_2_PO_4_, 25 NaHCO_3_, 20 glucose, 1.3 Na ascorbate and 0.6 Na pyruvate. Ringer solution consisted of (in mM) 125 NaCl, 2.5 KCl, 2 CaCl_2_, 1.3 MgCl_2_, 1.3 NaH_2_PO_4_, 25 NaHCO_3_, 10 glucose, 1.3 Na ascorbate and 0.6 Na pyruvate. For the whole-cell recording, the pipettes were filled with internal solution that contained (in mM) 130 K-gluconate, 10 HEPES, 0.6 EGTA, 5 KCl, 3 Na_2_ATP, 0.3 Na_3_GTP, 4 MgCl_2_ and 10 Na_2_-phosphocreatine (pH 7.2–7.4). Picrotoxin (50 μM) was added to the superfusion aCSF through the dilution of stock solutions. All chemicals for brain slicing recording were from Sigma-Aldrich. Whole-cell voltage-clamp recordings were performed with a MultiClamp 700B amplifier (Molecular Devices; USA). The neurons were clamped between −50 mV. The traces were low-pass filtered at 3 kHz and digitized at 10 kHz (DigiData 1440, Molecular Devices).

### Data analysis and statistical tests

No data were excluded. Photometry data were exported to MATLAB Mat files from Spike2 for further analysis. After smoothing the data with a moving average filter (20 ms span), we segmented the data based on behavioural events within individual trials or bouts. We derived the values of fluorescence change (Δ*F/F*) by calculating (*F*−*F*_0_)/*F*_0_, where *F*_0_ is the baseline fluorescence signal averaged over a 1.5-s-long control time window, which was typically set 0.5 s preceding the trigger events. For analysing the responses to mounting behaviour, the control time window was set 2.5 s before mounting onset to minimize the effect of active chasing. Δ*F/F* values were presented with heatmaps or average plots with a shaded area indicating s.e.m.

We used multivariate permutation tests to analyse the statistical significance of the event-related fluorescence change (ERF) or peri-event time histograms (PETHs) of spike firing rates as described elsewhere^68^. We used 1,000 permutations for an *α*-level of 0.05 to compare the values of *ΔF/F* or spike firing rate at each time point with the ERF or PETH baseline values. A series of statistical *P*-value at each time point were generated and the statistical results were superimposed on the average ERF or PETH curve with red and blue lines indicating statistically significant (*P*<0.05) increase or decrease, respectively.

To calculate the amplitude and activation duration of Δ*F/F* values, we first segmented the data based on the behavioural events of sucrose lick, food intake, mounting, male–male investigation or object investigation. The 95% fraction of the baseline Δ*F/F* values defined as the up threshold value and the 5% fraction as the low threshold. We then detected the local signal peaks during a given interaction bout using the MATLAB *findpeaks* function. The response amplitude of a behavioural bout was calculated by averaging all the local peaks above the up threshold value. The activation duration and inhibition duration was calculated by summing the time points above the up threshold or below the low threshold, respectively. The average bout peaks, activation duration and inhibition duration were averaged across the behavioural session to report the corresponding values of a given mouse. Wilcoxon's rank-sum test was applied to report the statistical significance of difference between mounting and male–male interaction. Non-parametric Wilcoxon's signed-rank test was performed to test the statistical significance of the difference between male–male interaction and object investigation.

PETH of spike firing rates (bin width 50 ms) were smoothed with a Gaussian kernel (*σ*=50 ms) and then presented either with heatmaps or average plots. To calculate *Z*-scores, we used the mean firing frequency of the entire trial instead of a control period, because most 5-HT neurons were silent (<1 spikes s^−1^) outside the reward zone. Hierarchical clustering was carried out in three steps. We first reduced the dimensionality of *Z*-scored firing activity via principle component analysis. The first three major principle components were then used to calculate Euclidean distance metric. The complete agglomeration method was finally applied to build the hierarchy of clusters and plot dendrograms in MATLAB.

Kolmogorov–Smirnov tests were carried out to evaluate the difference of trial durations, running times and poke latencies between training days in the one-arm foraging task. To examine the effects of reward omission, we first determined the peak values of the ERF or PETH data 2–3 s after the reward-zone-in event, when sucrose was delivered in rewarding trials. To examine the effect of reward delay, we measured the peak values of the ERF or PETH data at the point of sucrose delivery and the areas under the ERF or PETH curves during the delay phase (0–2 s and 0–5 s, respectively). Non-parametric Wilcoxon's signed-rank tests were performed to calculate statistical *P*-values.

## Additional information

**How to cite this article**: Li, Y. *et al*. Serotonin neurons in the dorsal raphe nucleus encode reward signals. *Nat. Commun*. 7:10503 doi: 10.1038/ncomms10503 (2016).

## Supplementary Material

Supplementary Information Supplementary Figures 1-9

Supplementary Movie 1 Sex activates 5-HT neurons.
