# Supplementary material for: Serotonin neurons in the dorsal raphe nucleus encode reward signals
Source: Nat Commun. 2016 Jan 28;7:10503. doi: 10.1038/ncomms10503 (PMC4738365; doi:10.1038/ncomms10503)
Supplement: Supplementary Information — Supplementary Figures 1-9 [file ncomms10503-s1.pdf]

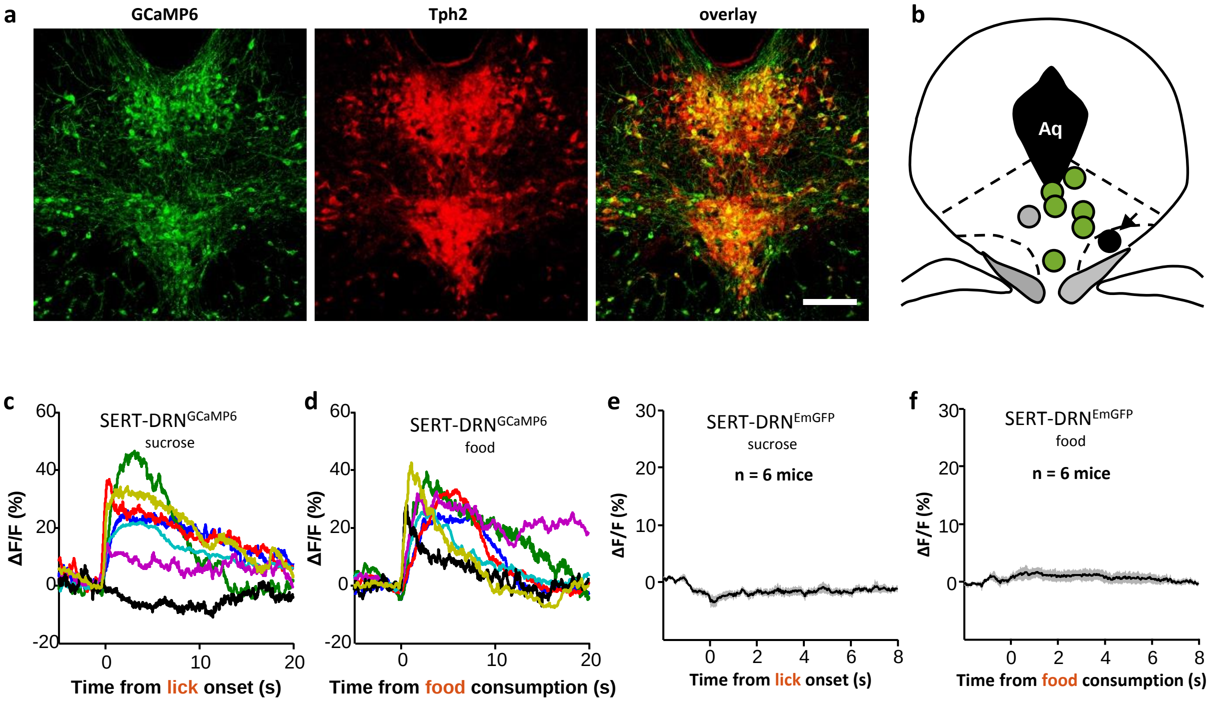

Supplementary Figure 1 | Sucrose lick and food intake evoke Ca^{2+} transients from DRN 5-HT neurons. (***a***) Colocalization of GCaMP6 (green) and tryptophan hydroxylase 2 (Tph2) immunoreactivity (red) in the DRN of a SERT-Cre mouse following the infusion of AAV-DIO-GCaMP6m virus. Of 1872 GCaMP6-expressing neurons, only 24 neurons were Tph2-negative (98.7% accuracy). Of 1873 Tph2-expressing neurons, only 25 neurons lacked clear GCaMP6 expression (98.7% efficiency). Scale bar = 200 μm. (***b***) Post mortem identification of the tip positions of optical fibers that were used to record the effect of sucrose lick and food intake. The dash lines delineate the DRN. Aq, aqueduct. The black circle (arrow) indicates the recording site that had weak GCaMP6 expression and lacked clear Ca^{2+} response to sucrose (black line in *c* and *d*). This mouse was not further studied in the sucrose foraging task and replaced by a mouse indicated with the grey circle. (***c*** and ***d***) Fluorescence intensity change of individual SERT-DRN^{GCaMP6} mice (n = 7) to sucrose lick (*c*) and food intake (*d*). (***e*** and ***f***) Mean GFP fluorescence aligned to sucrose lick onset (*e*) and food intake (*f*) in the SERT-DRN^{EmGFP} control mice (n = 6 mice). Thick lines indicate average and shaded areas indicate SEM.

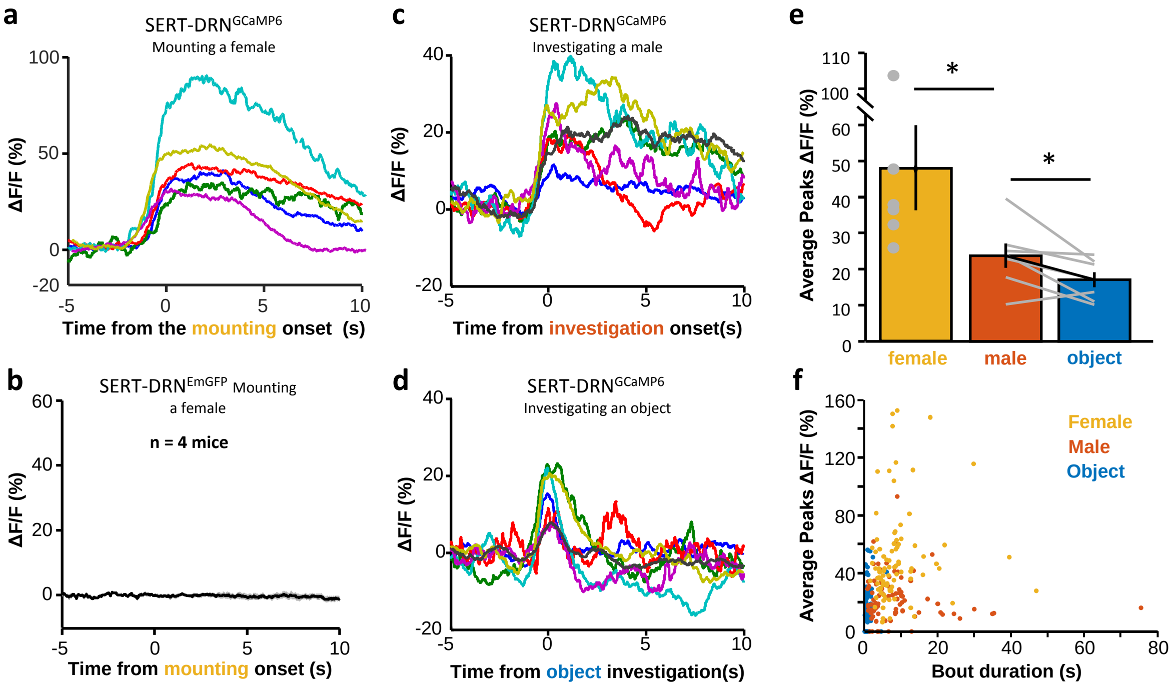

Supplementary Figure 2 | Ca^{2+} signals of 5-HT neurons related to social behaviors. (***a***) Ca^{2+} transients of individual male mice correlated with mounting a female. All behavioral bouts in a single session were averaged for individual mice (n = 6 SERT-DRN^{GCaMP6} male mice). (***b***) We did not observe any clear mating-associated fluorescence change from the EmGFP-expressing control mice (n = 4 SERT-DRN^{EmGFP} mice). (***c*** and ***d***) Calcium transients of the individual test mice follow their chemoinvestigation of another male (*c*) or investigation of a neutral object (dummy mouse; *d*). (***e***) Mounting a female evoked significantly stronger Ca^{2+} signals than investigation of another male (Wilcoxon rank sum test, p = 0.0221; n = 6 mice), which in turn produced larger responses than investigation of a neutral objet (Wilcoxon signed rank test, p = 0.0469; n = 7 mice). (*f*) Scatter plot of the peak Ca^{2+} amplitudes with the duration of behavioral bouts. The response amplitude was not significantly correlated with bout duration for both mating behavior (Pearson correlation coefficient r = 0.1256 , p = 0.25) or male-male interaction (r = - 0.1125, p = 0.31). Correlation between response amplitudes and investigation duration was mild but statistically significant (r = 0.2149, p = 0.04). Thick lines indicate average and shaded areas indicate SEM.

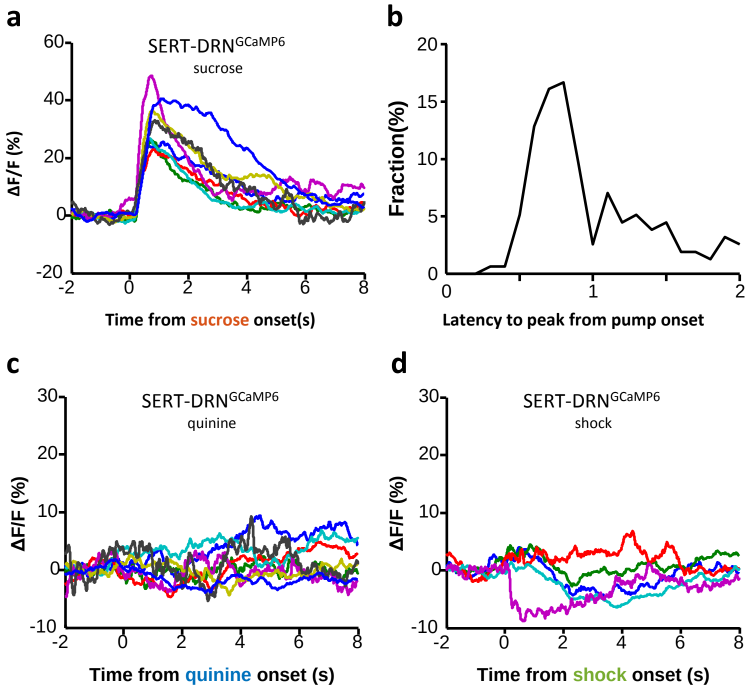

Supplementary Figure 3 | Ca^{2+} signals associated with random delivery of sucrose, quinine, and footshock. (***a***) The effect of random intraoral delivery of sucrose on the GCaMP signals in individual SERT-DRN^{GCaMP6} mice. Each line indicates the average responses of 20 deliveries to an individual mouse. (***b***) The distribution of latency to the peak of GCaMP signals (median = 0.84 s; 160 intra-oral deliveries from 8 mice). (***c***) The effect of random intraoral delivery of quinine solution (n = 8 mice). Same conventions as in (*a*). (***d***) Fluorescence intensity change of individual SERT-DRN^{GCaMP6} mice following random footshock (0.5 s; 0.7 mA). Each line represents the mean of 10 footshocks to an individual mouse (n = 5 mice).

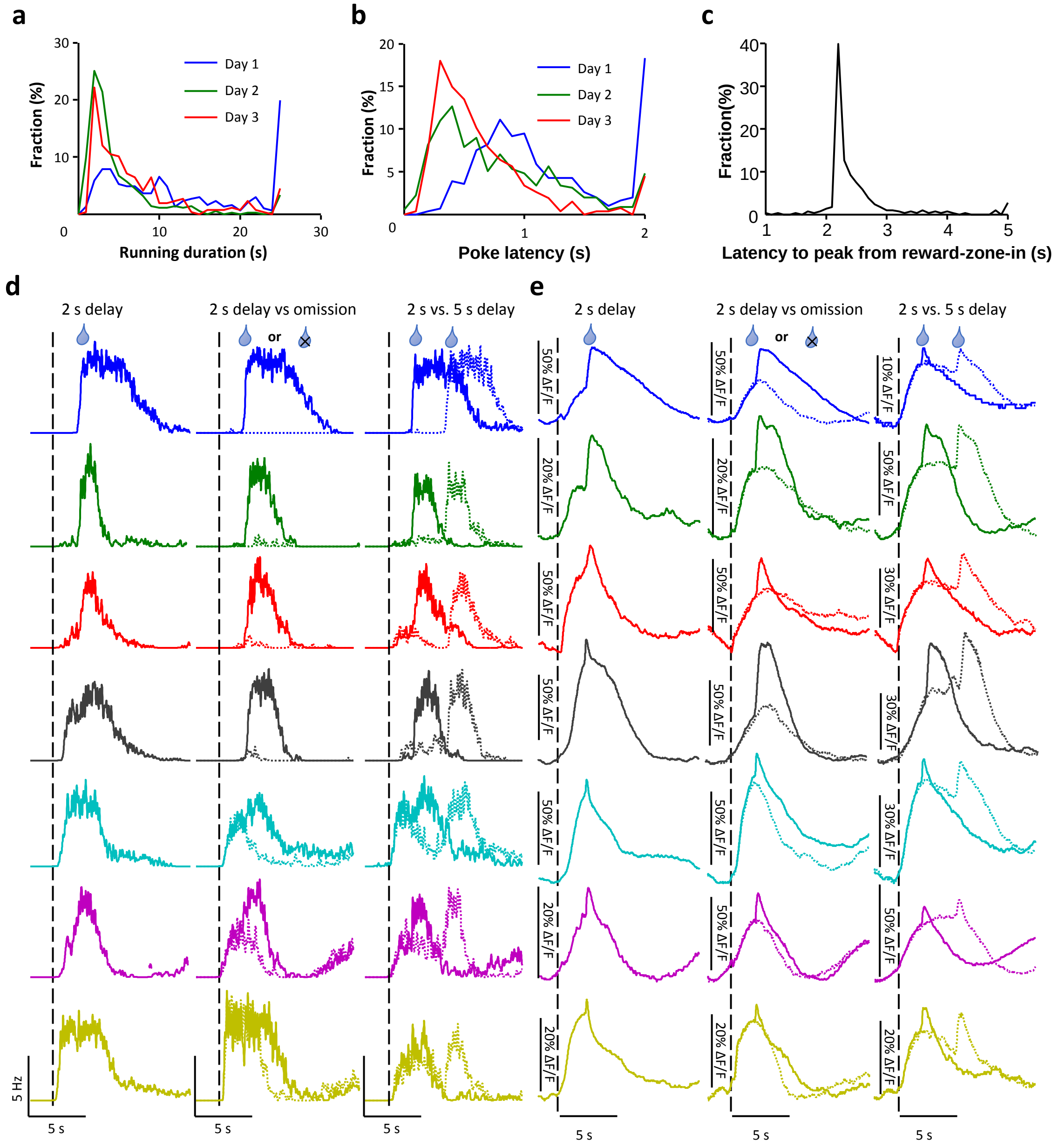

Supplementary Figure 4 | Ca^{2+} signals of DRN 5-HT neurons during the sucrose foraging task. (***a*** and ***b***) Behavioral characterization of the mice trained to forage for sucrose. (*a*) The distribution of running duration within the track linking the trigger zone and reward zone. Median of Day 1 = 10.2 s, Day 2 = 3.2 s, and Day 3 = 5.0 s. $p<0.001$ for Day 1 vs. Day 2, Day 1 vs. Day 3, and Day 2 vs. Day 3, Kolmogorov-Smirnov tests. (*b*) The distribution of nose poke latency following mouse entry into the reward zone. Median latency of Day 1 = 1.0 s, Day 2 = 0.6 s, and Day 3 = 0.5 s. $p<0.001$ for Day 1 vs. Day 2, Day 1 vs. Day 3, and Day 2 vs. Day 3, Kolmogorov-Smirnov tests. (***c***) The distribution of latency to the peak of GCaMP signals following mouse entry to the reward zone (median = 2.3; 294 trials in the 2 s-delay sessions of 7 mice). (***d***) Licking rates of the 7 individual mice engaged in sucrose foraging sessions with 2 s delay (left), sucrose delivery vs. sucrose omission (solid curves and dashed curves; middle), and 2 vs. 5 s delay (solid and dashed curves; right). Each row draws data from one SERT-DRN^{GCaMP6} mouse. Data are aligned to the reward-zone-in event (left vertical lines). The right dashed lines indicate the end of delay (2 s or 5 s). (***e***) Corresponding GCaMP signals of the 7 test mice in the behavioral tests of different reward outcomes and delays.

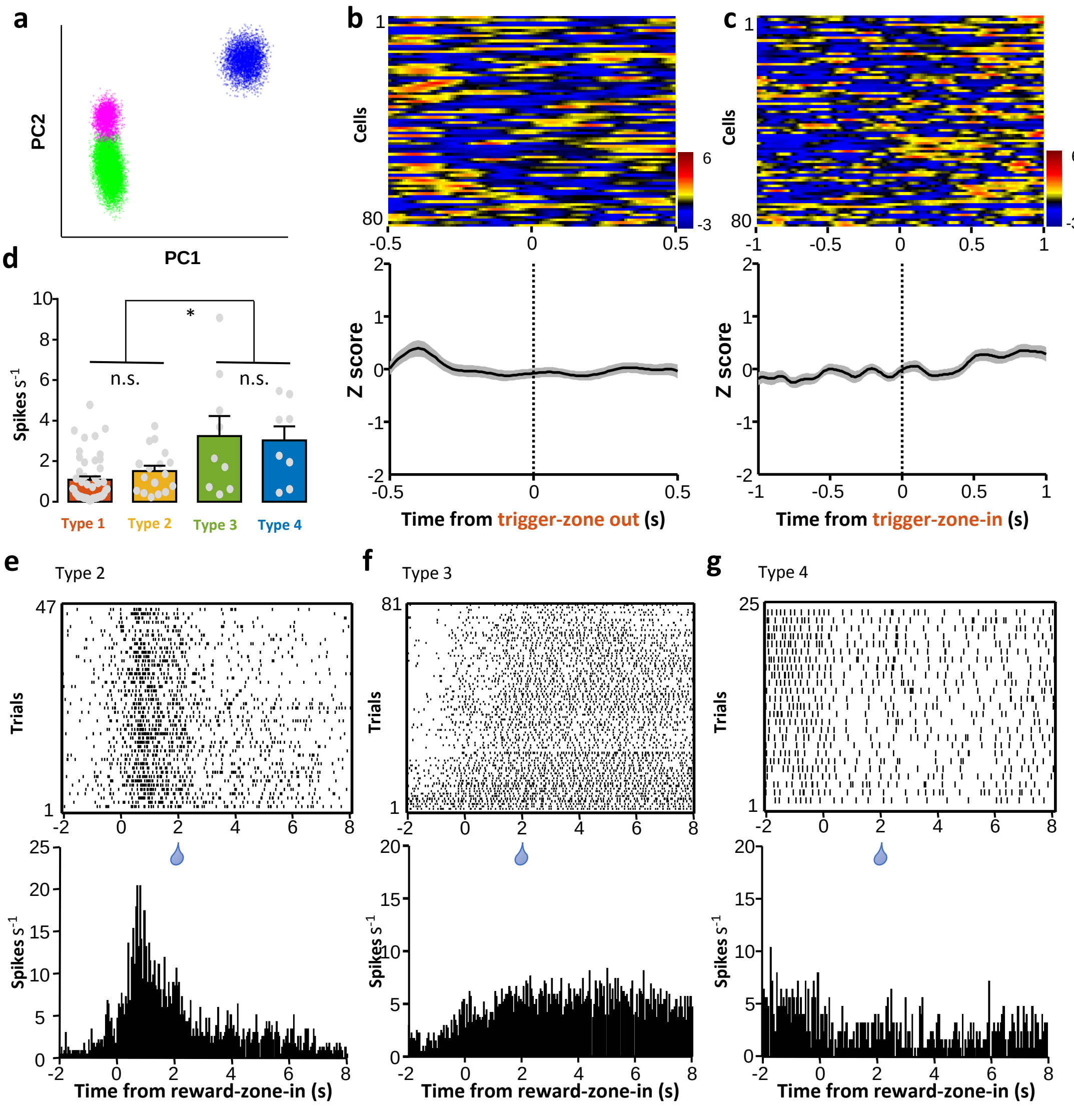

Supplementary Figure 5 | Spike firing patterns of 5-HT neurons during the sucrose foraging task. (***a***) Spike sorting using PCA. (***b*** and ***c***) Activity pattern of the 80 identified 5-HT neurons aligned to the trigger-zone-out event (*b*) and trigger-zone-in event (*c*). The upper panels show the heatmap representations of Z-scored PETH. Each row indicates one cell. The lower panels show the average Z scores. Thick lines indicate average and shaded areas indicate SEM. (***d***) Basal firing frequency of the 4 types tagged 5-HT neurons. *, $p < 0.05$, multiple comparisons after repeated measures one-way ANOVA. (***e***) Raster plot of spike firing pattern (upper panel) and the firing rate peri-event time histogram (PETH; lower panel) of a representative Type 2 5-HT neuron, which was activated during reward delay. The raster plot shows the spiking activity in 47 consecutive trials in a single behavior session. (***f***) The activity pattern of a typical Type 3 5-HT neuron, which mildly but continuously increased spike firing rate in a behavioral trial. (***g***) A typical Type 4 5-HT neuron, which exhibited a mild reduction of spike firing during the delay and following the delivery of sucrose solution.

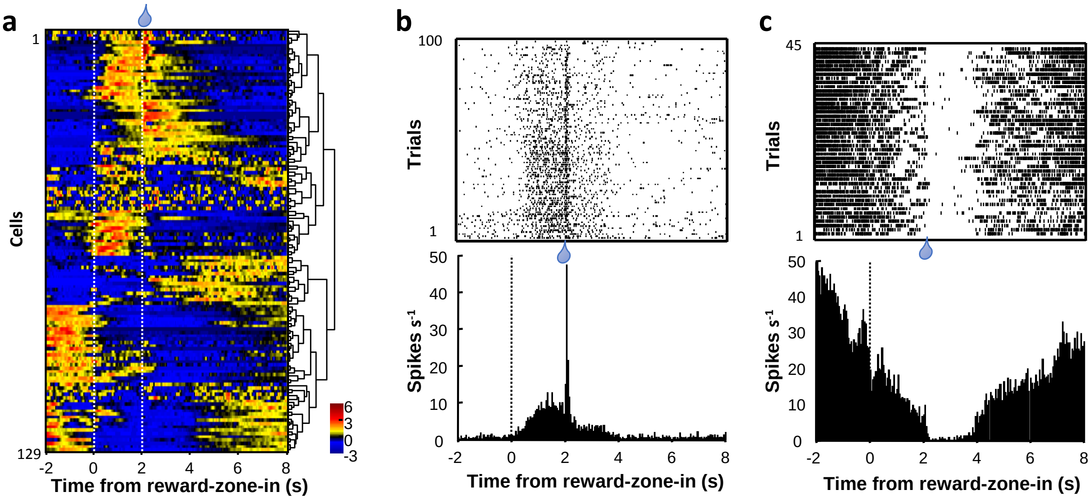

Supplementary Figure 6 | The activity patterns of DRN neurons of unidentified cell type information. (***a***) Heatmap representation of firing rate Z-scores of DRN neurons that were not confirmed with optical tagging (n = 129 non-tagged cells). Each line represents data from one cell. The dash lines indicate the reward-zone-in event and the delivery of sucrose solution, respectively. Data were hierarchically clustered based on the first three principal components of Z-scores (dendrogram at the right). **(*b*** and ***c*)** Two non-tagged DRN cells that showed either excitatory (***b***) or inhibitory responses (***c***) during reward seeking.

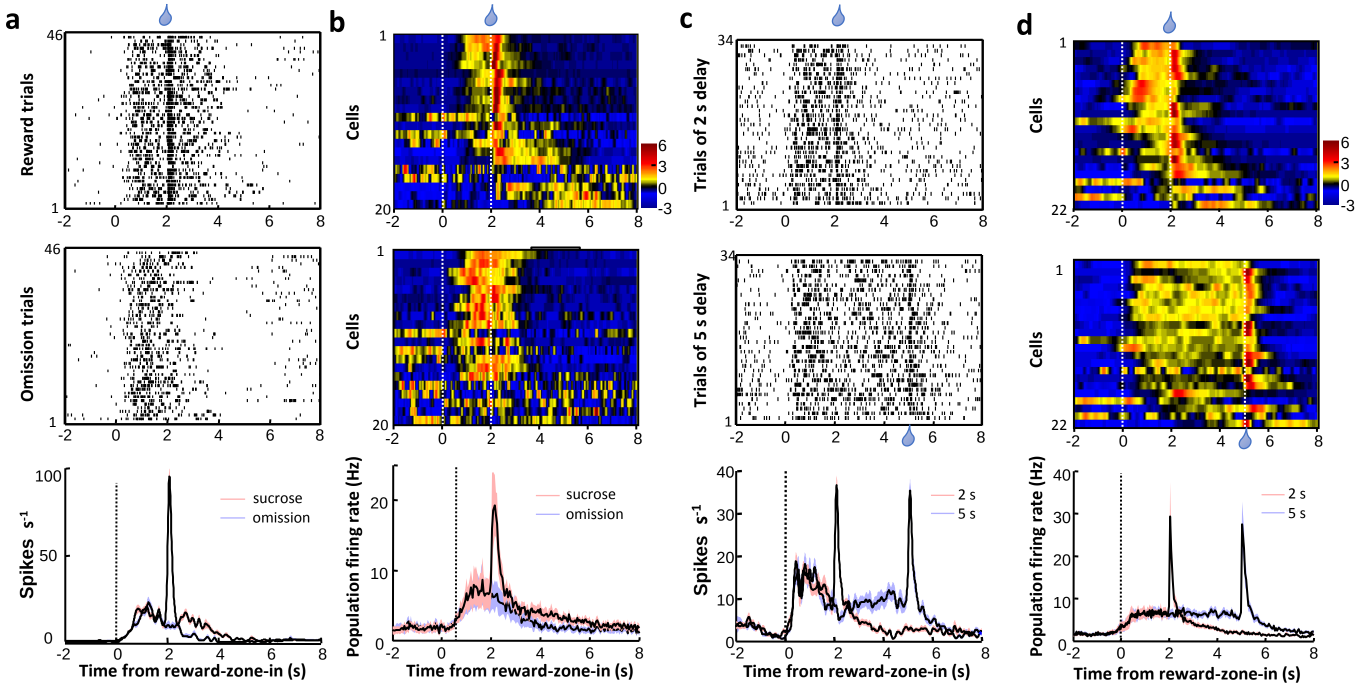

Supplementary Figure 7 | Effects of reward omission and delay on the activity pattern of DRN 5-HT neurons during the sucrose foraging task. (***a*** and ***b***) The effect of reward omission. (***a***) The raster plot of spike firing rate (upper) and PETHs (lower) of a representative 5-HT neuron that was tested with sucrose omission in half of trials. Reward trials and omission trials were randomly placed during the test and re-sorted offline. (***b***) The population data (n = 20 cells). The upper two panels in (***b***) show the heatmap representation of Z-scored PETHs. Cells were sorted in the descending order of response strength during 0.5 - 2.5s. The lower panel in (*b*) shows the group PETHs. (***c*** and ***d***) The effect of reward delay on the response of 5-HT neurons. (***c***) shows the response pattern of a representative 5-HT neuron and (***d***) shows the population responses (n = 22 cells). Thick lines indicate average and shaded areas indicate SEM.

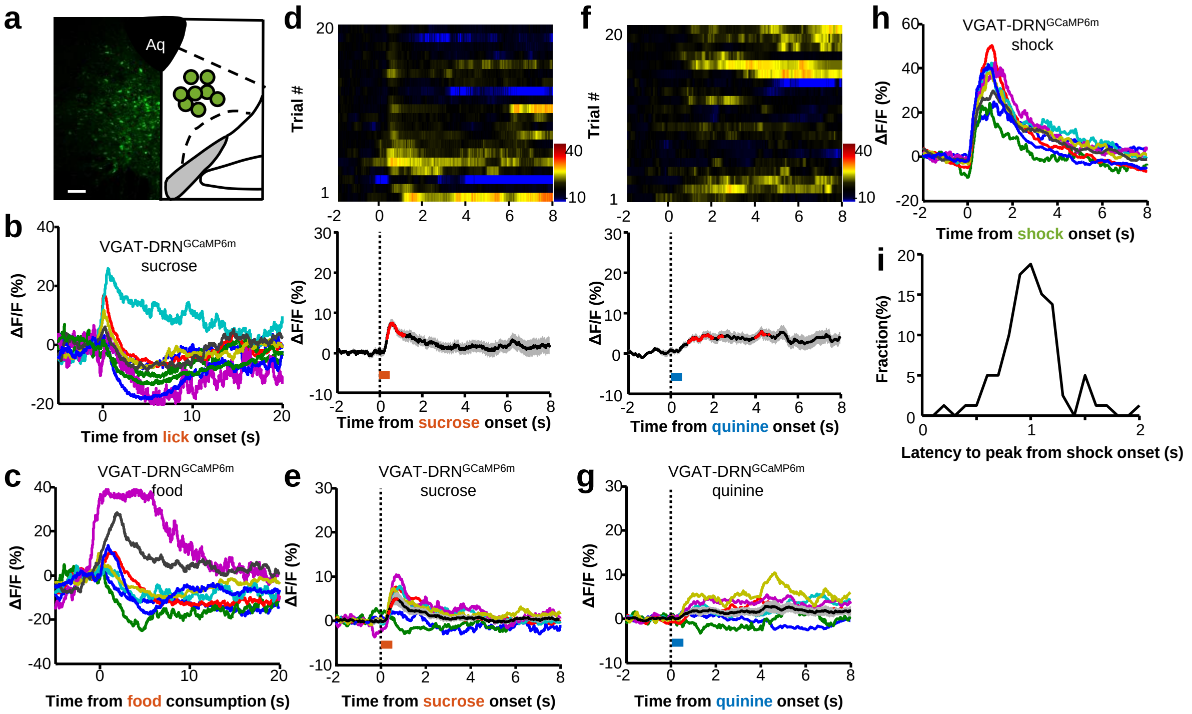

Supplementary Figure 8 | Ca^{2+} response profiles of DRN GABA neurons. (***a***) The location of optical fiber tip within the recorded VGAT-DRN^{GCaMP6m} mice (n = 8). Areas around the dash line represent DRN. Aq, aqueduct. Scale bar = 100 μm. (***b*** and ***c***) GCaMP fluorescence level changed when individual VGAT-DRN^{GCaMP6} mice voluntarily sought and acquired sucrose solution (*b*) and food (*c*). Each line indicates the average of all the bouts in a single session from individual mice. (***d*** and ***e***) Random intraoral delivery of sucrose produced very mild Ca^{2+} signals in a representative VGAT-DRN^{GCaMP6} mouse (*d*) and all test mice (*e*; n = 6). (***f*** and ***g***) Random intraoral delivery of quinine similarly produced small and variable Ca^{2+} signals in VGAT-DRN^{GCaMP6} mice. Same conventions as in (*d* and *e*). (***h***) Footshocks (0.5 s @ 0.7 mA) reliably elicited strong Ca^{2+} signals in all VGAT-DRN^{GCaMP6} test mice (n = 8). (***i***) Distribution of latency to the peak of GCaMP signals (median = 1.0 s; 80 trials by 8 mice). Thick lines indicate average and shaded areas indicate SEM.

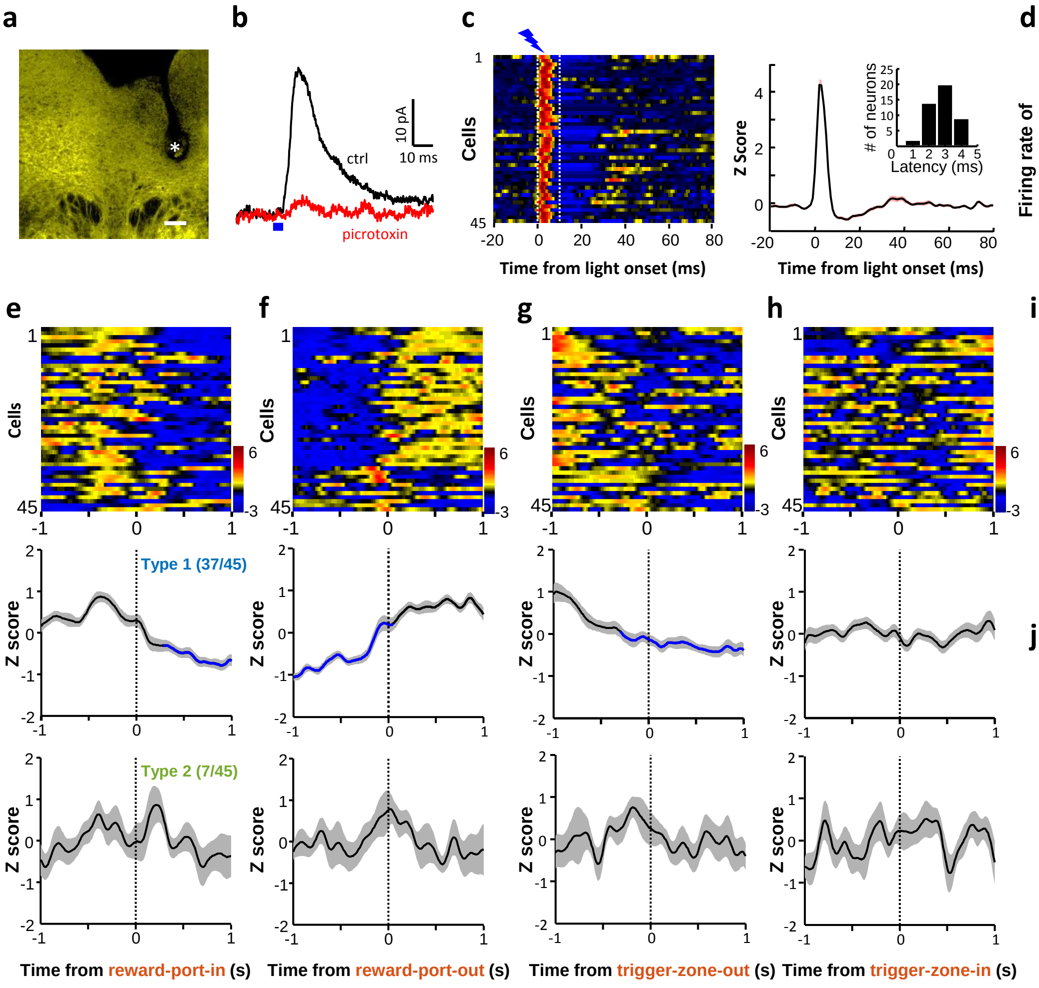

Supplementary Figure 9 | Spike firing patterns of DRN GABA neurons in freely behaving mice. (***a***) The tip location (asterisk) of an implanted optetrode in the DRN of a VGAT-ChR2 mouse. Scale bar = 100 µm. (***b***) Whole-cell recordings from DRN neurons in the slice preparation of VGAT-ChR2 mice (holding at -50 mV; n = 4 cells). Brief light pulses evoked an outward current that was blocked by the $GABA_A$ receptor antagonist picrotoxin (100 µM). **(*c*)** Optical tagging identified 45 GABA neurons from the DRN of behaving VGAT-ChR2-EYFP mice (n = 45 cells from 4 mice). Changes in neuronal firing rates were measured as Z scores. Neuronal activity change around the time of light stimulation (5 ms) was represented in the heatmap (left panel) and PETH (right panel). Inset in the right panel shows the latency distribution of light-evoked peak firing. (***d***) Scatter plot of the basal firing rates and the firing rate of maximum changes for identified 5-HT cells (red) and GABA cells (blue). Dashed line indicates no change. (***e-h***) Activity pattern of GABA neurons aligned to the reward-port-in event (*e*), the reward-port-out event (*f*), the trigger-zone-out event (*g*), and trigger-zone-in event (*h*). The top panels shows heatmap representation of the Z-scored PETHs of all 45 GABA cells. Cells were sorted in the same order as shown in Figure 8*c*. The middle and bottom panels show the average Z-scored PETH of Type 1 GABA neurons (middle) and Type 2 GABA neurons (lower panels). (***j***) Average firing rates of all identified GABA cells (n = 45 cells). Thick lines indicate average and shaded areas indicate SEM.
